# tRNA-derived fragments from wheat are potentially involved in susceptibility to Fusarium head blight

**DOI:** 10.1186/s12870-021-03393-9

**Published:** 2022-01-03

**Authors:** Zhengxi Sun, Yi Hu, Yilei Zhou, Ning Jiang, Sijia Hu, Lei Li, Tao Li

**Affiliations:** Key Laboratory of Plant Functional Genomics of the Ministry of Education/Jiangsu, Key Laboratory of Crop Genomics and Molecular Breeding/Collaborative Innovation of Modern Crops and Food Crops in Jiangsu/Jiangsu Key Laboratory of Crop Genetics and Physiology, College of Agriculture, Yangzhou University, Yangzhou, 225009 China

**Keywords:** Fusarium head blight, tRNA-derived small RNA fragments, RNase T2

## Abstract

**Background:**

Fusarium head blight (FHB) caused by *Fusarium graminearum* is a devastating fungal disease of wheat. The mechanism underlying *F. graminearum*-wheat interaction remains largely unknown. tRNA-derived fragments (tRFs) are RNase-dependent small RNAs derived from tRNAs, and they have not been reported in wheat yet, and whether tRFs are involved in wheat-*F. graminearum* interactions remains unknown.

**Results:**

Herein, small RNAs from the spikelets inoculated with *F. graminearum* and mock from an FHB-susceptible variety Chinese Spring (CS) and an FHB-resistant variety Sumai3 (SM) were sequenced respectively. A total of 1249 putative tRFs were identified, in which 15 tRFs was CS-specific and 12 SM-specific. Compared with mock inoculation, 39 tRFs were significantly up-regulated across both wheat varieties after *F. graminearum* challenge and only nine tRFs were significantly down-regulated. tRF^Glu^, tRF^Lys^ and tRF^Thr^ were dramatically induced by *F. graminearum* infection, with significantly higher fold changes in CS than those in SM. The expression patterns of the three highly induced tRFs were further validated by stem-loop qRT-PCR. The accumulation of tRFs were closely related to ribonucleases T2 family members, which were induced by *F. graminearum* challenge. The tRFs’ targets in host were predicted and were validated by RNA sequencing.

**Conclusion:**

Integrative analysis of the differentially expressed tRFs and their candidate targets indicated that tRF^Glu^, tRF^Lys^ and tRF^Thr^ might negatively regulate wheat resistance to FHB. Our results unvealed the potential roles of tRFs in wheat-*F. graminearum* interactions.

**Supplementary Information:**

The online version contains supplementary material available at 10.1186/s12870-021-03393-9.

## Background

Fusarium head blight (FHB), mainly caused by *Fusarium* species complex [1, 2], is one of the most devastating fungal disease of wheat. FHB epidemics can cause tremendous yield losses, and also have negative impacts on human and animal health due to mycotoxins deoxynivalenol (DON) contamination [3]. DON negatively regulates the protein synthesis by inhibiting the function of ribosome [4], and it also leads to cellular toxicity by inhibition of DNA and RNA synthesis [5], alteration of membrane structure [6], and by suppression of mitochondrial function and cell-cycle [7, 8].

Large number of quantitative trait loci (QTL) associated with FHB-resistance have been reported, and only two QTL (*Fhb1* and *Fhb7*) have been claimed to be cloned, however, their functions remain controversial [9–13], and our knowledge on the mechanism underlying wheat-*F. graminearum* interaction is still quite limited.

Transfer RNAs (tRNAs) are essential components of the translation machinery, and they also play roles in modulating cell proliferation and stress responses [14, 15]. tRFs can derive from tRNA precursor, but most are generated from cleavage of mature tRNAs [16, 17]. tRFs were classified into three types according to the region of cleavage and their size: long tRFs (circa 30-35 nt) originating from tRNA cleavage in the anticodon region, short tRFs (<28 nt) from cleavage either in the D or T region [18], and tiny tRFs (10-16 nt) [19]. Angiogenin, a kind of RNase A, is involved in the biogenesis of the long tRFs in human [20]. In yeast and protozoan, the long tRFs were produced through cleavage of tRNAs by Rny1p and Rnt2 respectively [21, 22]. Concerning short tRFs, DICER 1 or Dicer-like protein 1 (DCL1) was shown to cleave the D or T region of some tRNAs [17, 23–25]. However, RNases T2, instead of Dicer protein, was proposed to be involved in plant tRF biosynthesis [26]. The sizes of tiny tRFs range from 13 nt to 16 nt in Arabidopsis [19]. tRFs resemble the functional characteristics as microRNAs, including binding to AGO proteins, RISC complex formation with AGO proteins, and RNA silencing [24, 27–31]. The previous reports indicated that tRNAs were endonucleolytically cleaved under a variety of stress conditions [32]. tRFs were found in malignant human tumors and most of of them played pivotal functions in cancer progression and metabolic diseases [33–35]. tRFs firstly described in *Escherichia coli* responded to bacteriophage infection [36]. Recent report showed that RNase T2 was involved in the response to pathogen challenges [37]. Rhizobial tRNA-derived small RNAs were involved in cross-kingdom regulation of soybean nodulation [38]. tRFs in land plants were reported to participate in abiotic stress and development [19, 39, 40]. Until now, the databases about tRF in several plant species have been established, such as Arabidopsis, rice, soybean, maize, and grape (http://14.139.61.8/PtRFdb/index.php) [19, 41]. Wheat is one of the most important staple crops in the world, however, our knowledge on wheat tRFs is quite limited and the responses of wheat tRFs to *F. graminearum* challenge have not been reported yet.

In this study, through small RNA sequencing, tRFs in wheat under *F*. *graminearum* challenge and mock inoculation were examined; sizes and abundances of tRFs were analyzed; tRFs responsive to *F*. *graminearum* infection, their targets in host, and their potential functions in wheat-*F. graminearum* interaction were addressed. To our knowledge, this is the first report on tRFs in wheat responses to FHB. We hope the outcome of this study may provide a novel insight into the interactions between wheat and *F. graminearum*. Meanwhile, our data serves as a useful resource of wheat tRFs for further studying.

## Results

### Identification of tRFs in wheat spikelet by small RNA-seq

**Fig. 1:**
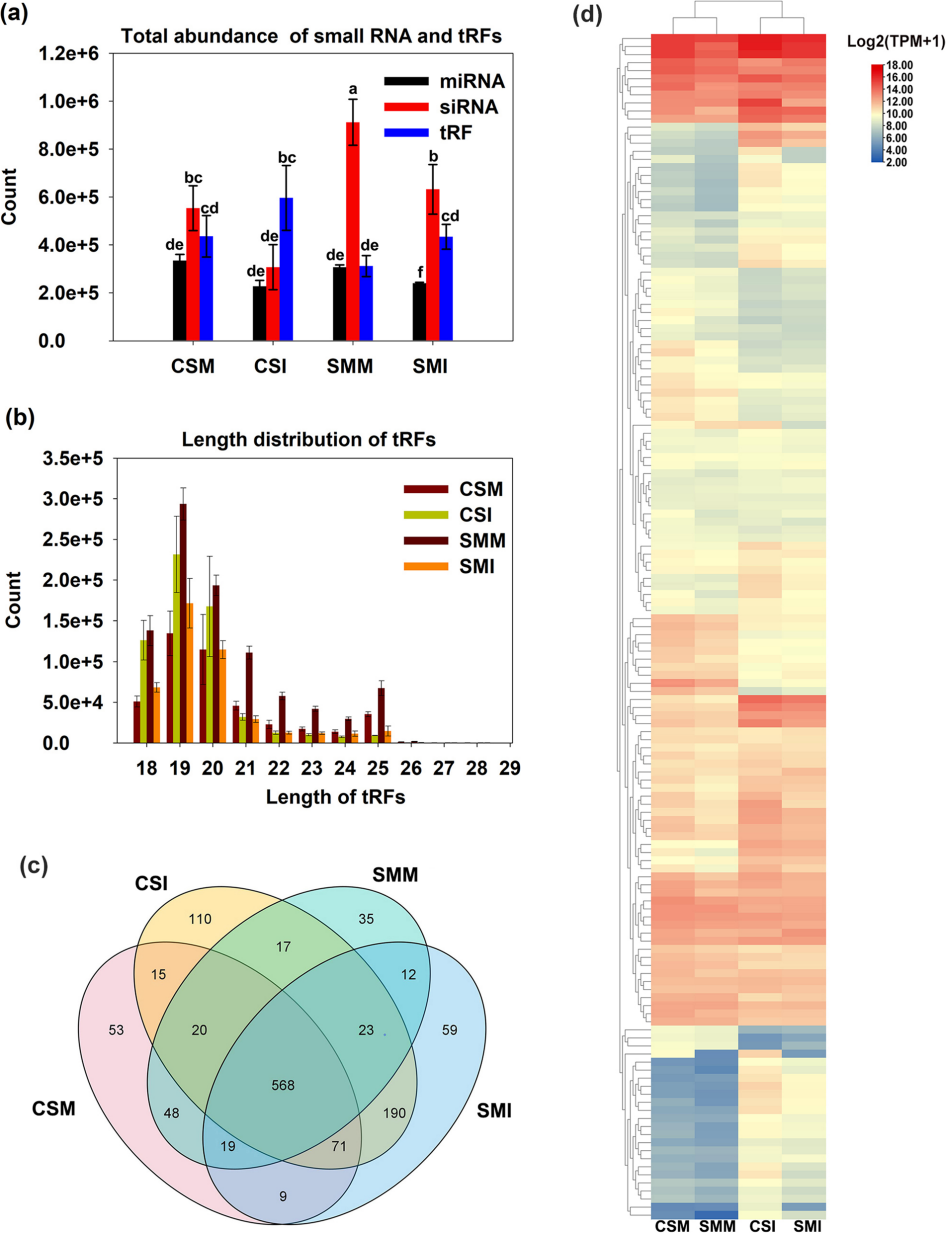
Summary of tRFs from sRNA-Seq. (**a**) Total counts of miRNA, siRNA and tRF in different groups. (**b**) Length distribution of tRFs. Count values are shown as means ± standard errors (s.e.) over three biological replicates. Different letters indicate significant differences according to the Student-Newman-Keulsa test (*p*<0.05). (**c**) Venn distribution of the identified tRFs among the four libraries. (**d**) Expression and cluster analysis of 147 highly expressed tRFs among the four libraries

Different responses to *F. graminearum* infection between CS and SM were observed, and only one or two symptomatic spikelets appeared on SM at 10 days post inoculation (dpi), whereas four or five symptomatic spikelets on CS (Fig. S1). Twelve libraries constructed from the total RNAs of the spikelets of CS and SM with *F. graminearum* infection and mock inoculation, respectively, were subjected to deep sequencing. Approximately 27 million raw tags of small RNA were generated in each library. At least 22 million clean tags in each library were mapped to the wheat genome. The Q20 of the clean tags were up to 99% (Table S1.1). The expressions of siRNAs were significantly downregulated by *F. graminearum* infection when compared with mock inoculation in both varieties, however*,* the expressional abundance of siRNAs in SM were significantly higher than their counterparts in CS across mock and *F. graminearum* inoculations (Fig. 1a). The number of tRFs was more than that of miRNAs under both *F. graminearum* and mock inoculations across two varieties, and the lengths predominantly ranged from 18 to 20 nt (Fig. 1a, b). Totally 1249 putative tRFs were identified, and all were derived from the tRNAs for transferring 20 types of amino acids, and the majority of them derived from the 5′end of the tRNAs (Table S2, Fig. S2). A total of 53, 110, 35 and 59 of specific tRFs were detected in CSM, CSI, SMM and SMI group respectively (Fig. 1c, Table S3), and 568 tRFs were in common across the twelve libraries, and 147 out of the 568 tRFs were enriched for at least 500 transcripts per million reads (TPM) in any of the four contrasting groups (Fig. 1c, Table S4). The heat map for the 147 tRFs clearly showed that tRF^Glu(CUC)^, tRF^Lys(CUU)^ and tRF^Thr(CGU)^ ranked top 3 in abundance both in CS and SM (Fig. 1d).

### Wheat tRFs were accumulated after infection by *F. graminearum* especially in FHB-susceptible variety

**Fig. 2:**
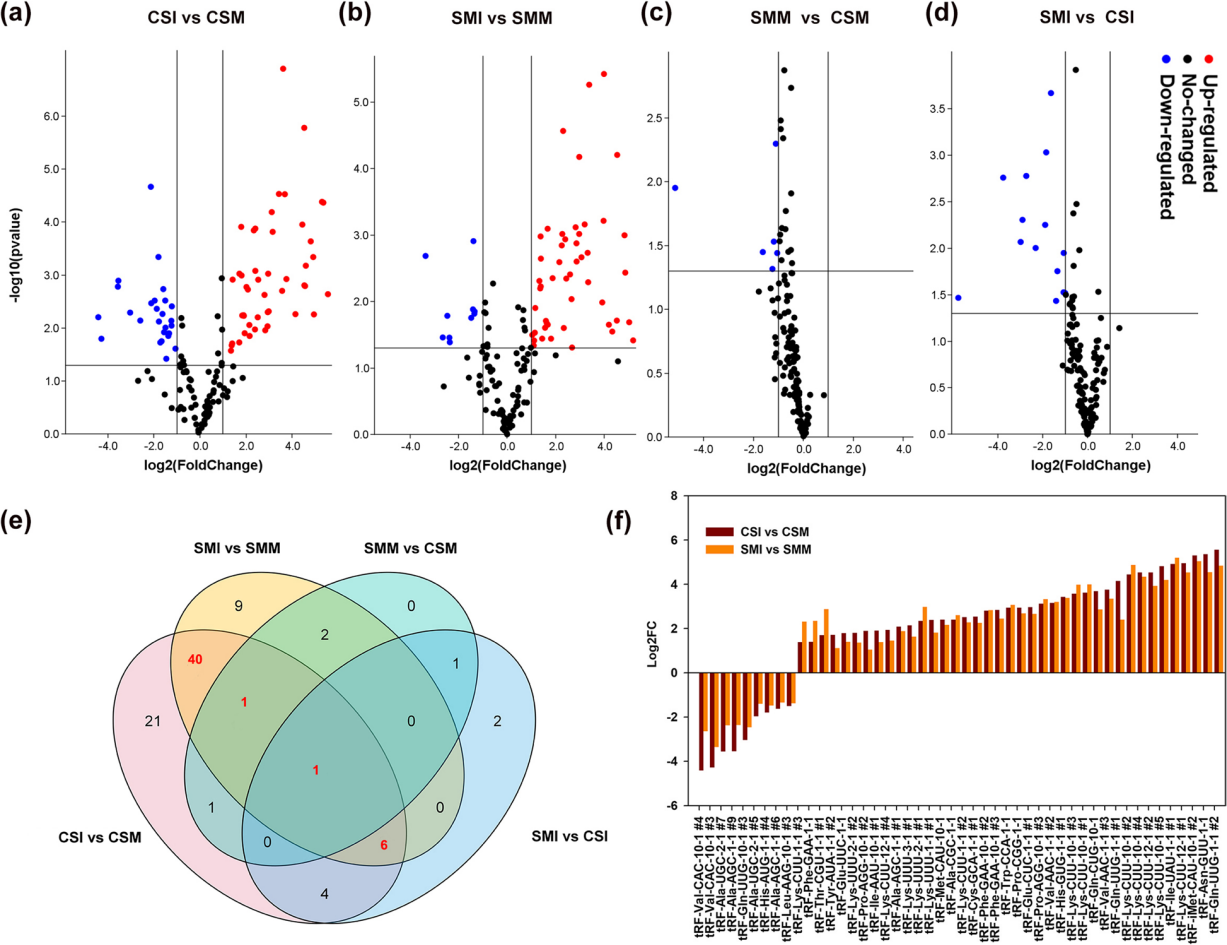
Differentially expressed wheat tRFs between *F. graminearum* and mock inoculation of CS and SM. Volcano plots show the comparisons between CSI and CSM (**a**), SMI and SMM (**b**), SMM and CSM (**c**), SMI and SMM (**d**). X-axis represents the Log2(fold change). Y-axis represents the *p* value in biological replication. The plots in red show the up-regulated genes, blue plots show the down-regulated genes, and blank plots show the unchanged genes. Each plot represents one gene. The significant differences were analyzed by *t* testing with the significance threshold of 0.05. (e) Venn diagram shows the distribution of differentially expressed tRFs among comparisons of the four libraries. Red in bold represents the number of the tRFs associated with *F. graminearum* infection. (f) Fold changes of 48 differentially expressed tRFs in comparisons of CSI-vs-CSM and SMI-vs-SMM

Responsive patterns of tRFs to *F. graminearum* and mock inoculation were compared between CSI and CSM, and between SMI and SMM. Seventy-four tRFs had significant differences in abundance between CSM and CSI, with 47 tRFs being upregulated and 27 downregulated after *F. graminearum* infection (Fig. 2a). Fifty-nine tRFs had significant differences in abundance between SMM and SMI, with 49 tRFs being upregulated and 10 downregulated after *F. graminearum* infection (Fig. 2b). We also compared the expressional patterns of tRFs between the two varieties, SMM versus CSM, and SMI versus CSI. CS accumulated more tRFs across the two contrasting inoculations (Fig. 2c, d). It is surprising that all of the significantly differentially expressed (*p*< 0.05) tRFs appeared only in CS (Fig. 2c, d). Forty-eight significantly differentially expressed tRFs were associated with *F. graminearum* infection, with tRF^Gln(UUG)^ and tRF^Val(CAC)^ showing largest changes (Fig. 2e, f). Interestingly, 14 of the 48 tRFs were derived from tRNA-Lys (Table 1). Moreover, all of the 14 tRF^Lys^ were dramatically accumulated by *F. graminearum* infection both in CS and SM, but the abundances in CS were higher than those in SM. tRFs derived from tRNA-Lys-CUU-6-1 were CS-specific (Table S3.5). Interestingly, one tRNA, tRNA-Lys-CUU-10-1, produced diversiform short fragments. The tRFs primarily derived from the 5′ end of the tRNAs, and only five tRFs derived from the 3′ end of the tRNAs (Table S5, Fig. S2). The majority of the tRFs varied from 18 to 21 nt in length, and only a few tRFs had size up to 25 nt, such as tRF^Ala(AGC)^ and tRF^Cys(GCA)^ (Fig. S3).

**Table 1:** Differentially expressed tRFs between CS and SM after *F. graminearum* (CSI and SMI) or mung bean broth (CSM and SMM) inoculation

tRF ID	Sequence	Length	CSM	CSI	SMM	SMI	Log2 (CSI/CSM)	Log2 (SMI/SMM)
tRF-Ala-AGC-1-1 #1	GCGAGAGGTACGGGGATCG	19	132	557	73	267	2.08	1.88
tRF-Ala-AGC-1-1 #6	GGGGATGTAGCTCAGATGGTAG	22	3470	1122	2600	1020	-1.63	-1.35
tRF-Ala-AGC-1-1 #9	GGGGATGTAGCTCAGATGGTAGAGC	25	7892	676	4697	914	-3.54	-2.36
tRF-Ala-CGC-1-1	GCGAGAGGCACGGGGTTCG	19	198	1048	108	654	2.40	2.60
tRF-Ala-UGC-2-1 #5	GGGGATGTAGCTCAAATGGTAG	22	1849	472	1534	578	-1.97	-1.41
tRF-Ala-UGC-2-1 #7	GGGGATGTAGCTCAAATGGTAGAGC	25	3663	309	2472	475	-3.57	-2.38
tRF-Asn-GUU-1-1	AACCACAAGGTCGGAGGT	18	36	1493	41	963	5.36	4.54
tRF-Cys-GCA-1-1 #1	GGGTCCATAGCTCAGTGG	18	1888	10909	1762	8410	2.53	2.26
tRF-Gln-CUG-10-1	TCCAGTAACCCGAGTTCA	18	90	1155	51	373	3.69	2.86
tRF-Gln-UUG-10-1 #3	GGTTTCGTAGTGTAGTGGTTAG	22	689	84	565	102	-3.04	-2.46
tRF-Gln-UUG-1-1 #1	TCTGGCGACCTGGGTTCG	18	78	1389	53	279	4.15	2.40
tRF-Gln-UUG-1-1 #2	ATCTGGCGACCTGGGTTCG	19	43	2029	35	1014	5.56	4.84
tRF-Glu-CUC-1-1 #1	CCGTCGTAGTCTAGGTGG	18	410	3198	292	1842	2.96	2.66
tRF-Glu-UUC-1-1	TCCATTGTCGTCCAGCGG	18	827	2866	1000	2629	1.79	1.40
tRF-His-AUG-1-1 #4	GACAGTTTGGCCGAGTGGTCT	21	758	217	725	260	-1.80	-1.48
tRF-His-GUG-1-1 #1	GCCGTGGAGACCTGGGCT	18	1592	17168	1167	12197	3.43	3.39
tRF-Ile-AAU-10-1 #1	GGCCTATTAGCTCAGCTG	18	515	1929	432	1125	1.91	1.38
tRF-Ile-UAU-1-1 #1	CTCCCGTAGCTCAGTTGG	18	26	778	8	306	4.92	5.20
tRF-iMet-CAU-10-1 #2	TAACCCACAGGTCCCAGGATCG	22	43	1698	31	1006	5.30	5.04
tRF-Leu-AAG-10-1 #3	GATCAGATGGCCGAGTTGGT	20	2410	847	1979	757	-1.51	-1.39
tRF-Lys-CUU-10-1 #1	CCTTGTGGTCGTGGGTTC	18	79	971	42	675	3.61	4.00
tRF-Lys-CUU-10-1 #2	CCTTGTGGTCGTGGGTTCG	19	69	1514	32	948	4.45	4.87
tRF-Lys-CUU-10-1 #3	AACCTTGTGGTCGTGGGTTC	20	84	994	44	688	3.57	3.98
tRF-Lys-CUU-10-1 #4	AACCTTGTGGTCGTGGGTTCG	21	301	6962	213	4317	4.53	4.34
tRF-Lys-CUU-10-1 #5	TAACCTTGTGGTCGTGGGTTCG	22	57	1612	51	930	4.81	4.20
tRF-Lys-CUU-1-1 #2	GCCCGTCTAGCTCAGTCGG	19	900	5128	849	4114	2.51	2.28
tRF-Lys-CUU-1-1 #3	GCCCGTCTAGCTCAGTCGGT	20	8242	21424	3721	18462	1.38	2.31
tRF-Lys-CUU-12-1 #1	CCCGTCTAGCTCAGTTGG	18	41	1279	21	484	4.95	4.53
tRF-Lys-CUU-12-1 #4	GCCCGTCTAGCTCAGTTGG	19	2763	10617	2112	5761	1.94	1.45
tRF-Lys-CUU-12-1 #2	CCCGTCTAGCTCAGTTGGT	19	230	5333	167	2542	4.54	3.93
tRF-Lys-UUU-1-1 #1	GCCGTCTTAGCTCAGTTGG	19	154	807	123	432	2.39	1.81
tRF-Lys-UUU-2-1 #1	CCGACCTAGCTCAGTGGT	18	174	883	103	806	2.34	2.97
tRF-Lys-UUU-2-1 #2	GCCGACCTAGCTCAGTGG	18	1611	5643	1438	3708	1.81	1.37
tRF-Lys-UUU-3-1 #1	GCCGTCCTAGCTCAGTTGG	19	302	1335	265	823	2.15	1.64
tRF-Met-CAU-10-1	TCCTGAGGTCGAGAGTTC	18	331	1750	256	1139	2.40	2.16
tRF-Phe-GAA-10-1 #2	TCTGAAGGTCGCGTGTTCG	19	200	1396	123	873	2.80	2.83
tRF-Phe-GAA-10-1 #3	ATCTGAAGGTCGCGTGTTCG	20	95	676	82	447	2.84	2.44
tRF-Phe-GAA-1-1	GCGGGGATAGCTCAGTTG	18	424	1119	226	1145	1.40	2.34
tRF-Pro-AGG-10-1 #2	GCGAGAGGTCCCGAGTTC	18	354	1317	413	848	1.89	1.04
tRF-Pro-AGG-10-1 #3	GTGCGAGAGGTCCCGAGT	18	74	640	75	750	3.12	3.32
tRF-Pro-CGG-1-1	GCGAGAGGTCCCGAGTTCG	19	175	1348	125	799	2.95	2.68
tRF-Thr-CGU-1-1 #1	CCTCCGTAGCATAGTGGT	18	1256	4100	507	3699	1.71	2.87
tRF-Trp-CCA-1-1	TCAGAAGGTTGCGTGTTCG	19	192	1468	110	920	2.94	3.07
tRF-Tyr-AUA-1-1 #2	CCGACCTTAGCTCAGTTGG	19	5560	18222	5276	11434	1.71	1.12
tRF-Val-AAC-1-1 #2	ACTGAAGGTCTCCGGTTCG	19	59	524	46	420	3.16	3.20
tRF-Val-AAC-1-1 #3	CTGAAGGTCTCCGGTTCG	18	66	892	61	620	3.76	3.34
tRF-Val-CAC-10-1 #3	GTCTGGGTGGTGTAGTTGGTTAT	23	630	32	542	53	-4.28	-3.36
tRF-Val-CAC-10-1 #4	GTCTGGGTGGTGTAGTTGGTTATC	24	755	35	593	94	-4.42	-2.65

“tRF ID” originates from the tRNA name of wheat tRNA database (http://gtrnadb.ucsc.edu/GtRNAdb2/). The values in CSM, CSI, SMM and SMI columns were the TPM (Transcript per million reads) values over three replicates.

**Fig. 3:**
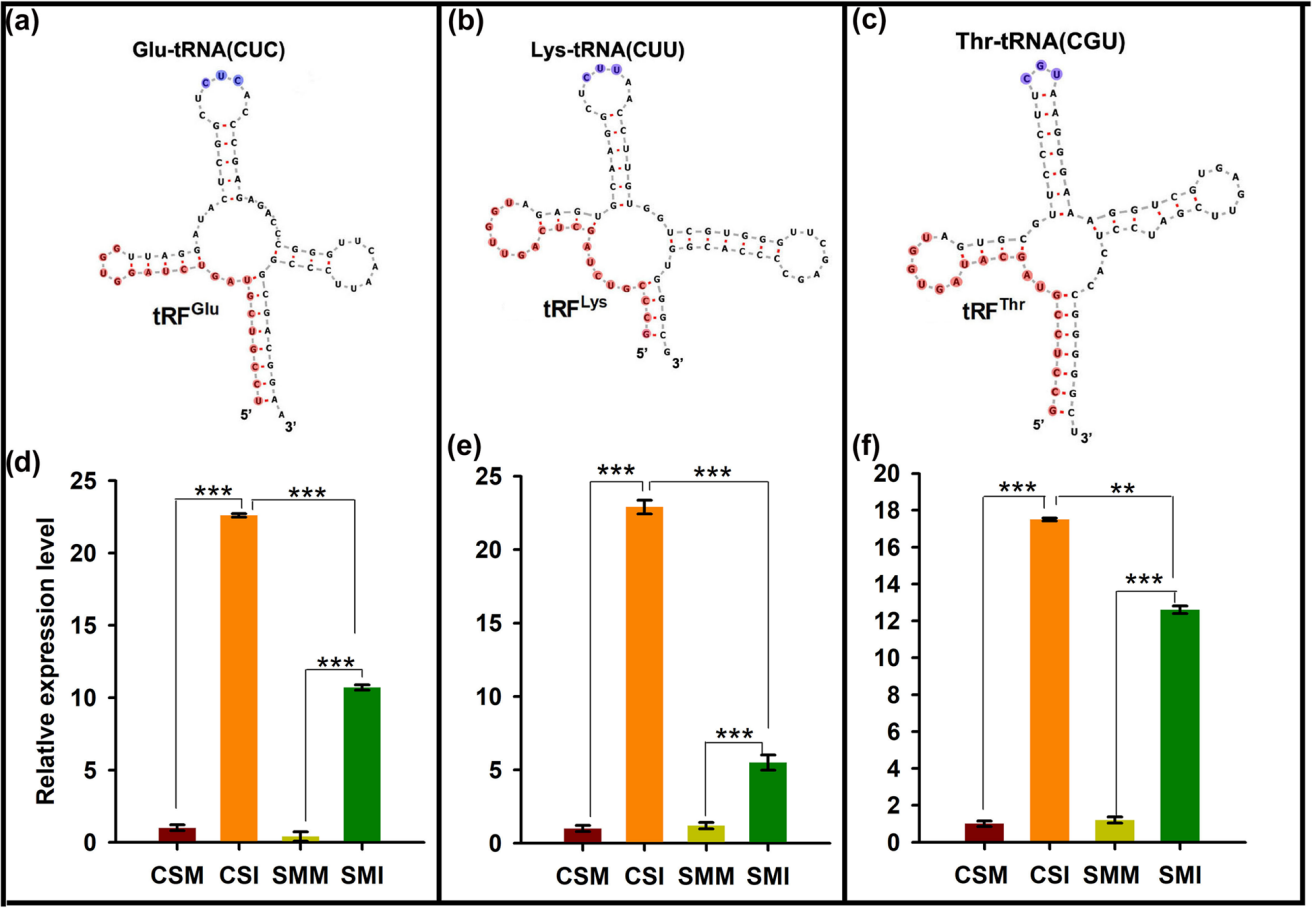
Three highly induced wheat tRFs and their expressional patterns. Precursors of tRF^Glu^ (**a**), tRF^Lys^ (**b**) and tRF^Thr^ (**c**). Anticodons in corresponding tRNAs are colored in blue; The sequences of tRFs were marked in red color. (d), (e) and (f) show the expressional levels of corresponding tRFs by stem-loop-qRT-PCR. The snRNA gene *TaU6* was used as a loading control. Three independent biological repeats showed similar results. RNAfoldWebServer was used to draw the tRNA primary structures of tRFs. Student’s *t*-test was used for significant difference, ***, *P* < 0.001; **, *P* < 0.01

Clone sequencing of the top three tRFs in abundance, tRF^Glu(CUC)^, tRF^Lys(CUU)^ and tRF^Thr(CGU)^, validated the tRFs from small RNA sequencing (Supplemental Data 1). The primary structures of tRNA indicated that all the three tRFs derived from the 5’ end of tRNAs (Fig. 3a, b, c). Stem-loop qRT-PCR validated the differentially expressed tRFs from small RNA-Seq, and these three tRFs were dramatically induced by *F. graminearum* in both varieties, but CS accumulated significantly more tRFs than those in SM (Fig. 3d, e, f). At same time, we checked the expressions of these three tRFs at different time points (Fig. S4). tRF^Glu^ was induced at 2 days post *F. graminearum* inoculation (dpi) in CS, but at 3 dpi in SM, and the degree of induction in CS was significantly higher than that in SM at 6 dpi (Fig. S4a). tRF^Lys^ was significantly induced at 2 dpi and 5 dpi in CS, whereas was slightly induced at 5 dpi in SM (Fig. S4b). tRF^Thr^ was induced at 12 h after inoculation with *F. graminearum* in both varieties, but was strongly induced at 2 dpi in SM. At later stages of *F. graminearum* infection, tRF was significantly induced only in CS (Fig. S4c). In short, the expression patterns of all these three tRFs reflect a common rule, that is, they were induced in CS, and the induction amplitude gradually increased with the temporal infection progression.

### RNase T2 was closely associated with the formation of tRFs

**Fig. 4:**
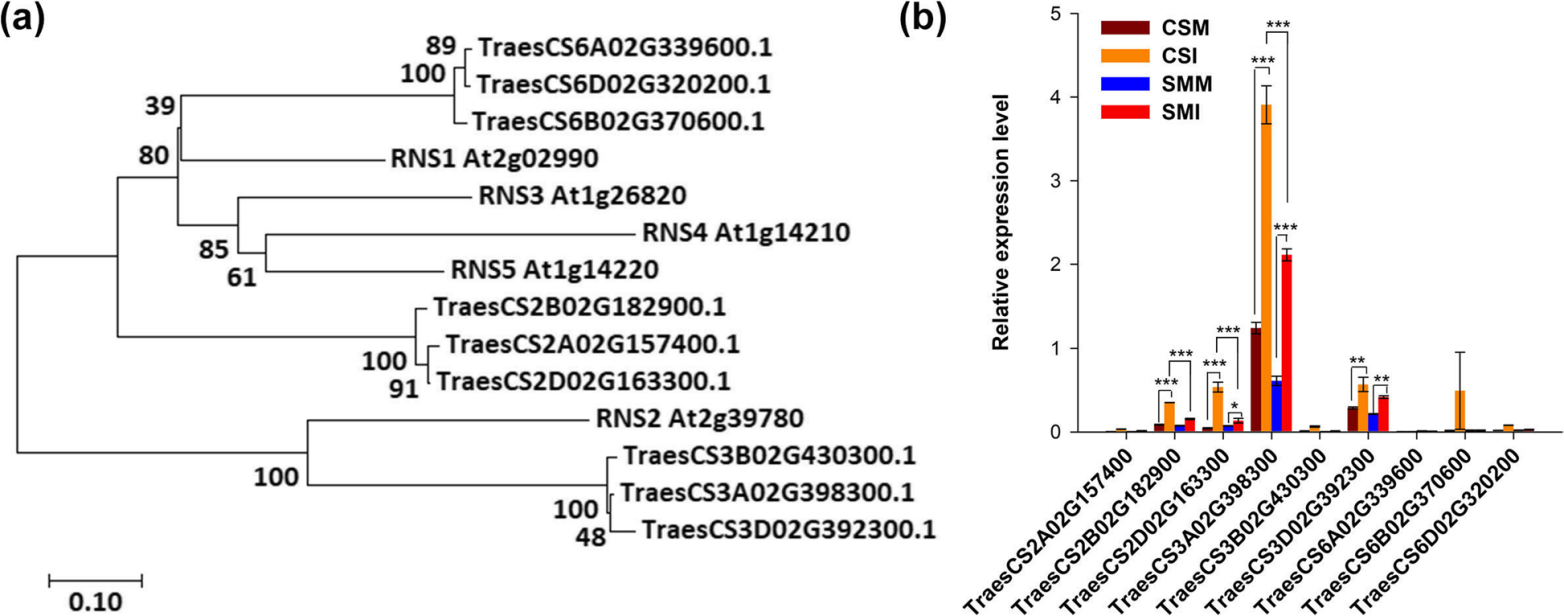
*RNaseT2* family members in wheat. (**a**) Phylogenetic tree of RNAase T2 proteins in wheat and Arabidopsis. The Neighbor-Joining (NJ) tree was generated using MEGA7 with 1000 bootstrap replicates. (**b**) Expressional detection of *TaRNase T2* family members by qRT-PCR. Expressional values are shown as means ± standard errors (s.e.) over three replicates. Expressional levels are calculated in 2^-ΔΔCt^. The *TaActin* was used as a loading control. Student’s *t*-test was used for significant difference, ***, *P* < 0.001; **, *P* < 0.01; *, *P* < 0.05

To understand the association of RNase T2 with tRF formation and accumulation, the changes of wheat RNase T2 family members were analyzed under *F. graminearum* and mock inoculations. S-like RNase genes *RNS1*, *RNS2*, *RNS3* and *RNS4* are RNase T2 genes in Arabidopsis. Blasting RNS1, RNS2, RNS3 and RNS4 against wheat genome database identified nine wheat RNase T2 orthologues, which were located in Chrs.2A/2B/2D, Chrs.3A/3B/3D and Chrs.6A/6B/6D, respectively (Fig. 4a). Four of them (*TraesCS2B02G182900*, *TraesCS2D02G163300*, *TraesCS3A02G398300* and *TraesCS3D02G392300*) were significantly induced by *F*. *graminearum* challenge of CS, and their intensities of induction in CS were significantly higher than their counterparts in SM (Fig. S5, Fig. 4b). There were no significant changes or only low expressional levels for the other five RNase T2 genes, suggesting that not all RNase T2 members were induced by *F. graminearum* to cleave the accumulated tRNAs in wheat. Notably, *TraesCS3A02G398300* was significantly induced and might be the main producer of tRFs (Fig. 4b). Additionally, we analyzed the expressions of *TaAGO1* and *TaDCL* family members in our transcriptome data. Surprisingly, all of *TaAGO1* and *TaDCL* family members were significantly inhibited by *F. graminearum* across two wheat varieties (Figure S6). These results further illustrated that RNase T2 probably played a pivotal role in tRF biosynthesis.

### Prediction and validation of tRFs’ targets in wheat

**Fig. 5:**
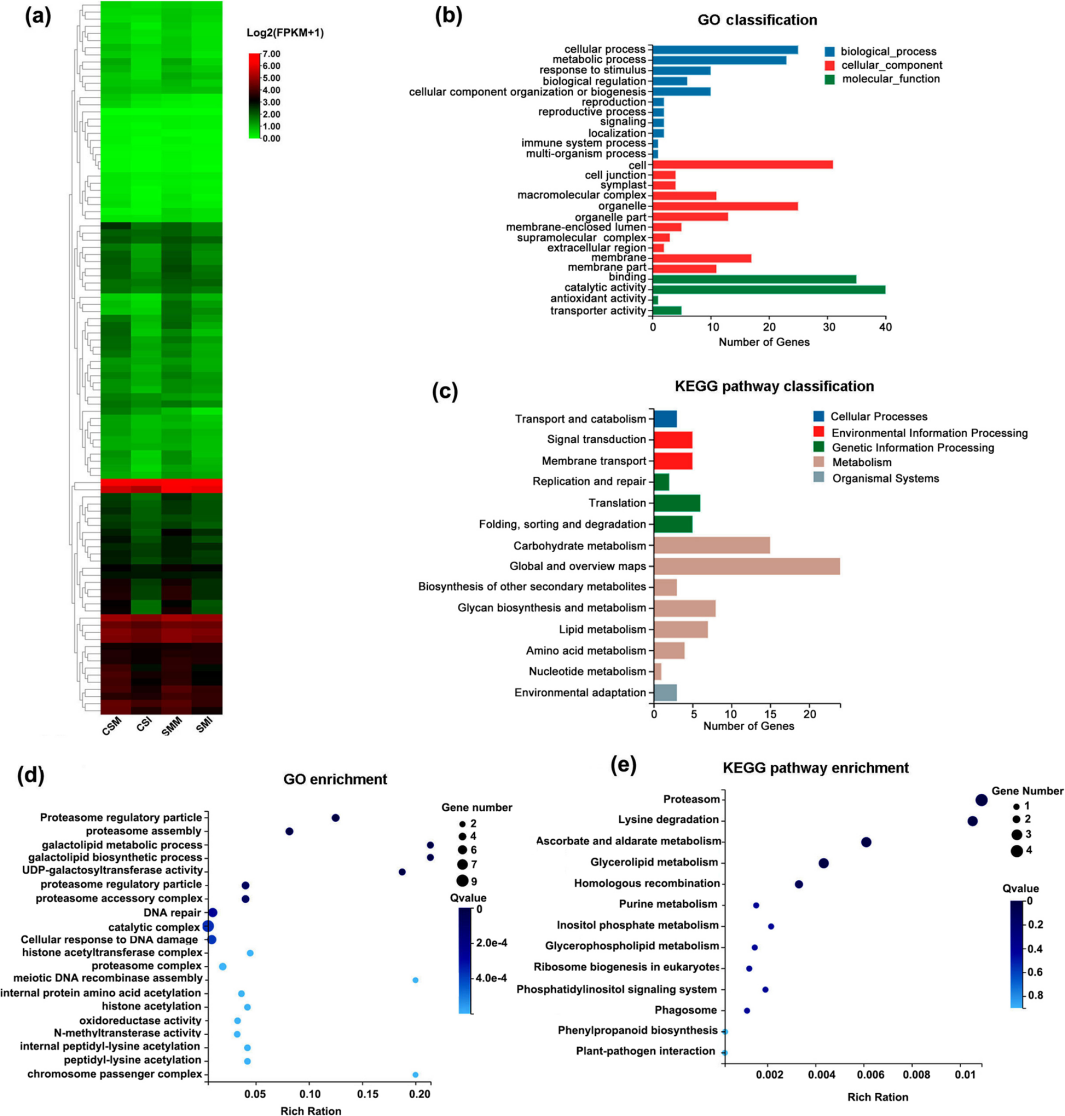
Analyses of expression, GO and KEGG of differentially expressed tRFs’ target genes predicted in wheat. (**a**) Heatmap of expressions of differentially expressed tRFs’ target genes. The value is normalized from the average expression level for each row using ZeroToOne scale method. (**b**) GO classification analysis of the 100 predicted tRFs’ target genes. (**c**) KEGG pathway classification of the 100 predicted tRFs’ targets. (**d**) Scatter plot of GO enrichment of the 100 predicted tRF targets. (e) Scatter plot of KEGG pathway enrichment of the 100 predicted tRFs’ targets. The enrichment ratio is measured by the number of the genes to the number of background genes in a particular GO term or KEGG pathway. The size of the dots represents the number of genes, and the color of the dots represents the range of the Q-value

Twelve mRNA libraries corresponding to the 12 small RNA libraries were constructed to identify the potential tRFs’ targets. An average of 120 million raw reads of mRNA was generated in each library, and at least 103 million clean reads in each library were mapped to the wheat genome. Q20 of the clean reads was up to 96% (Table S1.2). Up to 100 target genes of high confidence for all the identified tRFs were predicted in wheat (Table S6). Notably, almost all of the predicted targets in CS and SM were downregulated after infection by *F*. *graminearum*, and the extent of downregulation was stronger in CS than that in SM (Fig. 5a). The expressional levels of the tRFs’ targets were negatively associated with the expressions of the tRFs (Table 2), suggesting tRFs might inhibit the expressional level of their targets as miRNAs’ action. Gene Ontology (GO) classification showed that the terms “cellular process”, “metabolic process” and “response to stimulus” were dominantly enriched in the biological process category. In the cellular component ontology, “cell”, “organelle” and “membrane” were the highly abundant categories. The genes dramatically enriched in the molecular function category were involved in “catalytic activity” and “binding” (Fig. 5b). The most enriched GO terms were those associated with the proteasome components or its organization, followed by galactolipid biosynthetic and metabolic process (Fig. 5d). DNA damage and repair related terms were also significantly enriched. Kyoto Encyclopedia of Gene and Genomes (KEGG) pathway analysis showed “metabolism pathway” was the most represented pathway, including “carbonhydrate metabolism”, “biosynthesis of other secondary metabolites”, “glycan biosynthesis and metabolism”, “lipid metabolism”, “amino acid metabolism” and “nucleotide metabolism”, and most of these genes were classified into “carbonhydrate metabolism” pathway (Fig. 5c). “ascorbate and aldarate metabolism” and “lysine degradation” were also significantly enriched (Fig. 5e). The KEGG pathway enrichment analysis supported the results from the GO enrichment analysis, such as “proteasome”, “glycerolipid metabolism” and “purine metabolism”.

**Table 2:** The predicted targets of highly induced tRFs by *F*. *graminearum*

tRF	Target_Acc.	Exp.	log2(CSI/CSM)	*p* value	log2(SMI/SMM)	*p* value	Annotation
tRF^Ala(CGC)^	TraesCS6D02G244300	2.5	-0.45	0.5722	-0.25	0.2465	Dual specificity protein kinase YAK1
tRF^Ala(CGC)^	TraesCS4B02G385200	2.5	-1.09	0.0553	-1.05	0.0370	Homeobox-leucine zipper protein
tRF^Ala(UGC)^	TraesCS2A02G097900	2.5	-0.71	0.0304	-0.24	0.2946	DNA-directed primase/polymerase protein
tRF^Ala(UGC)^	TraesCS2B02G113600	2.5	-0.80	0.0752	-0.92	0.0008	DNA-directed primase/polymerase protein
tRF^Cys(GCA)^	TraesCS7B02G203400	2	-2.64	0.0024	-1.93	0.0025	ABC transporter B family member 19-like
tRF^Cys(GCA)^	TraesCS7D02G298600	2	-2.26	0.0432	-1.25	0.0038	ABC transporter B family member 19-like
tRF^Cys(GCA)^	TraesCS6B02G170700	2	-0.68	0.0001	-0.57	0.0133	ABC transporter B family member 19-like
tRF^Cys(GCA)^	TraesCS6D02G131900	2	-1.76	0.0045	-1.07	0.0029	ABC transporter B family member 19-like
tRF^Glu(CUC)^	TraesCS6B02G032300	2.5	-1.34	0.0015	-0.68	0.1562	Pentatricopeptide repeat-containing protein
tRF^Glu(CUC)^	TraesCS6D02G026900	2.5	-0.82	0.0046	-0.69	0.0073	Pentatricopeptide repeat-containing protein
tRF^Glu(CUC)^	TraesCS3A02G085700	2.5	-0.75	0.0286	-0.35	0.3106	Trehalose 6-phosphate phosphatase
tRF^Glu(CUC)^	TraesCS3D02G085800	2.5	-0.77	0.0572	-0.95	0.0630	Trehalose 6-phosphate phosphatase
tRF^Glu(CUC)^	TraesCS6D02G395700	2.5	-0.93	0.1064	-0.48	0.1659	Interleukin-1 receptor-associated kinase 4
tRF^Glu(CUC)^	TraesCS5A02G002500	2.5	-2.40	0.0101	-0.64	0.2172	1-phosphatidylinositol-4-phosphate 5-kinase
tRF^Glu(CUC)^	TraesCS5D02G002700	2.5	-2.47	0.0567	-1.29	0.0146	1-phosphatidylinositol-4-phosphate 5-kinase
tRF^His(AUG)^	TraesCS7B02G416600	2	-1.51	0.0241	-0.17	0.6981	Disease resistance protein RPM1
tRF^His(AUG)^	TraesCS6A02G222000	2.5	-0.40	0.1980	-0.64	0.0465	Phytosulfokine receptor 1
tRF^His(AUG)^	TraesCS6B02G291900	2.5	-1.37	0.0137	-0.97	0.0527	ABC transporter B family member 2-like
tRF^iMet(CAU)^	TraesCS2B02G598800	2.5	-0.88	0.0015	-0.79	0.0010	Polyadenylate-binding protein 4-like
tRF^iMet(CAU)^	TraesCS2B02G045400	2.5	-0.50	0.0429	-0.49	0.0229	Disease resistance RPP13-like protein 1
tRF^iMet(CAU)^	TraesCS2A02G030000	2.5	-1.18	0.0081	-0.91	0.0028	Disease resistance RPP13-like protein 1
tRF^iMet(CAU)^	TraesCS2B02G044900	2.5	-0.79	0.0047	-0.49	0.0058	Disease resistance RPP13-like protein 1
tRF^iMet(CAU)^	TraesCS2A02G029800	2.5	-0.43	0.0097	-0.34	0.0460	Disease resistance RPP13-like protein 1
tRF^iMet(CAU)^	TraesCS2D02G337300	2.5	-0.28	0.0164	-0.09	0.5289	Translation initiation factor 4B
tRF^iMet(CAU)^	TraesCS2A02G325400	2.5	-0.27	0.0781	-0.15	0.2779	Translation initiation factor 4B
tRF^Leu(AAG)^	TraesCS3B02G590000	2	-0.24	0.0213	-0.26	0.0180	UDP-D-apiose/UDP-D-xylose synthase
tRF^Lys(CUU)^	TraesCS6A02G164200	2	-0.37	0.0065	-0.24	0.0506	Phosphatidylcholine transfer protein SFH2-like
tRF^Lys(CUU)^	TraesCS1D02G264300	2.5	-0.71	0.0127	-0.45	0.0158	Ubinuclein
tRF^Lys(CUU)^	TraesCS6D02G254900	2.5	-1.31	0.0004	-1.06	0.0001	Histone-lysine N-methyltransferase SUVR5
tRF^Lys(CUU)^	TraesCS6A02G274600	2.5	-1.11	0.0007	-1.05	0.0001	Histone-lysine N-methyltransferase SUVR5
tRF^Lys(CUU)^	TraesCS6B02G302100	2.5	-1.94	0.0010	-1.45	0.0006	Histone-lysine N-methyltransferase SUVR5
tRF^Lys(CUU)^	TraesCS6D02G041400	2.5	-1.43	0.1912	-1.23	0.1284	Disease resistance protein RPS2
tRF^Lys(CUU)^	TraesCS6B02G341700	2.5	-1.00	0.0183	-0.70	0.0056	TORTIFOLIA1-like protein 3
tRF^Lys(CUU)^	TraesCS4D02G211200	2.5	-0.43	0.0111	-0.45	0.0164	Digalactosyldiacylglycerol synthase
tRF^Lys(CUU)^	TraesCS4A02G093900	2.5	-0.71	0.0031	-0.53	0.0124	Digalactosyldiacylglycerol synthase
tRF^Lys(CUU)^	TraesCS4B02G210400	2.5	-1.74	0.0005	-1.21	0.0002	Digalactosyldiacylglycerol synthase
tRF^Lys(CUU)^	TraesCS1A02G341400	1	-1.04	0.2031	-0.31	0.5059	E3 ubiquitin-protein ligase
tRF^Lys(CUU)^	TraesCS1D02G343700	2.5	-1.55	0.1171	-0.38	0.4265	E3 ubiquitin-protein ligase
tRF^Lys(CUU)^	TraesCS1B02G354100	0	-1.33	0.0733	-0.94	0.0969	E3 ubiquitin-protein ligase
tRF^Lys(UUU)^	TraesCS4B02G253500	2.5	-1.39	0.0016	-0.93	0.0001	Alpha-L-fucosidase 2
tRF^Lys(UUU)^	TraesCS4B02G253300	2.5	-0.80	0.0027	-0.66	0.0004	Alpha-L-fucosidase 2
tRF^Lys(UUU)^	TraesCS4D02G253400	2.5	-1.05	0.0008	-0.74	0.0028	Alpha-L-fucosidase 2
tRF^Lys(UUU)^	TraesCS4A02G051000	2.5	-0.80	0.0068	-0.76	0.0018	Alpha-L-fucosidase 2
tRF^Pro(CGG)^	TraesCS5A02G096600	0.5	-0.19	0.3818	-0.12	0.5391	Mitochondrial carrier protein
tRF^Pro(CGG)^	TraesCS7B02G437300	2.5	-0.53	0.0923	-0.54	0.0429	Disease resistance protein RPM13
tRF^Thr(CGU)^	TraesCS2B02G167000	1.5	-1.31	0.0001	-1.18	0.0008	Transcription-associated protein
tRF^Thr(CGU)^	TraesCS2A02G141800	1.5	-1.40	0.0001	-1.18	0.0005	Transcription-associated protein
tRF^Thr(CGU)^	TraesCS2D02G145300	1.5	-1.77	0.0001	-1.47	0.0012	Transcription-associated protein
tRF^Thr(CGU)^	TraesCS3D02G364200	2.5	-0.94	0.0004	-0.74	0.0011	WD40 domain contain protein
tRF^Thr(CGU)^	TraesCS3A02G371100	2.5	-0.78	0.1390	-0.62	0.5027	WD40 domain contain protein
tRF^Thr(CGU)^	TraesCS1B02G075900	2.5	-0.87	0.2030	-0.33	0.6064	DNA repair protein RAD51 like protein
tRF^Thr(CGU)^	TraesCS1D02G059600	2.5	-0.97	0.4057	-0.33	0.6472	DNA repair protein RAD51 like protein
tRF^Trp(CCA)^	TraesCS5D02G256900	2.5	-0.66	0.0037	-0.33	0.0131	tRNA (cytosine(34)-C(5))-methyltransferase
tRF^Trp(CCA)^	TraesCS5B02G247600	2.5	-0.82	0.0083	-0.43	0.0256	tRNA (cytosine(34)-C(5))-methyltransferase
tRF^Tyr(AUA)^	TraesCS4A02G406400	0	-0.49	0.0106	-0.50	0.0318	Dynein assembly factor 1
tRF^Tyr(AUA)^	TraesCS1B02G371300	2.5	-0.43	0.3945	-0.39	0.3175	Alpha-1,3-arabinosyltransferase XAT2-like
tRF^Val(AAC)^	TraesCS1D02G283000	1.5	-0.73	0.3259	-1.41	0.2318	Laccase
tRF^Val(AAC)^	TraesCS7B02G408700	2	-2.33	0.1853	-0.56	0.2495	ARR transcriptional factor
tRF^Val(AAC)^	TraesCS7B02G194500	2.5	-0.82	0.0113	-0.37	0.1357	Galactan beta-1,4-galactosyltransferase GALS1
tRF^Val(AAC)^	TraesCS4A02G444300	2.5	-0.89	0.0013	-0.75	0.0003	Phosphoglucan, water dikinase

The targets of tRFs were analyzed using psRNATargets analysis server (http://plantgrn.noble.org/psRNATarget/analysis). The list of tRF sequences were submitted and the cDNA library of *Triticum aestivum* for target search.

**Fig. 6:**
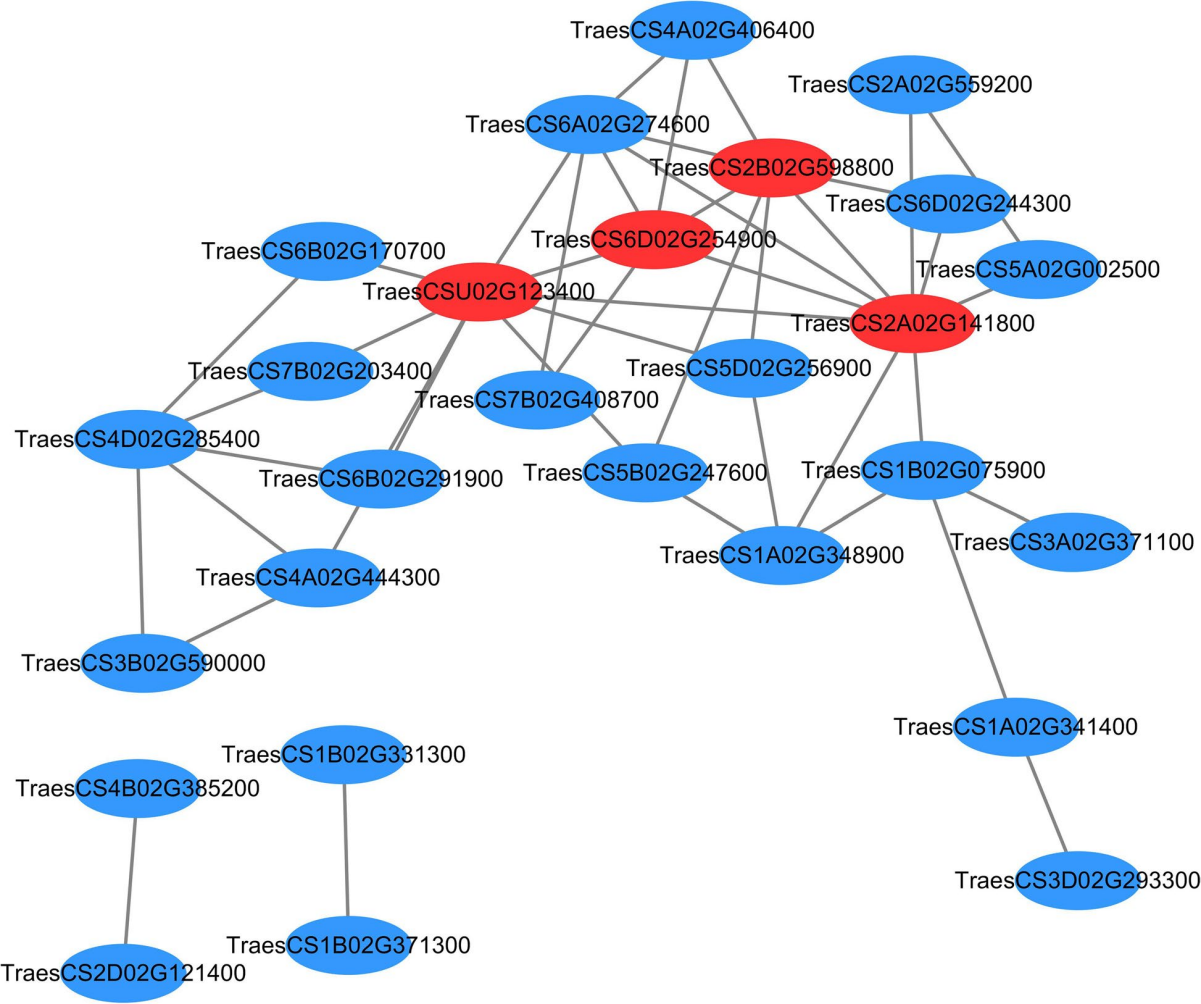
The correlation networks among all of the differentially expressed target genes. Candidate hub targets are shown in red ellipses. STRING (version 11) was used to analyze the interaction between the target genes. Blue ellipses represent proteins of the corresponding wheat genes. The two ellipses connected by the gray line represent the interaction between the proteins

In order to capture the key tRFs targets, protein interaction analysis was performed. Four hub tRFs targets were uncovered, *TraesCS2B02G598800*, *TraesCS6D02G254900*, *TraesCSU02G123400* and *TraesCS2A02G141800* (Fig. 6)*,* which were the candidate target genes of tRF^iMet(CAU)^, tRF^Lys(CUU)^, tRF^Ile(GAU)^and tRF^Thr(CGU)^, respectively (Table S6).

**Fig. 7:**
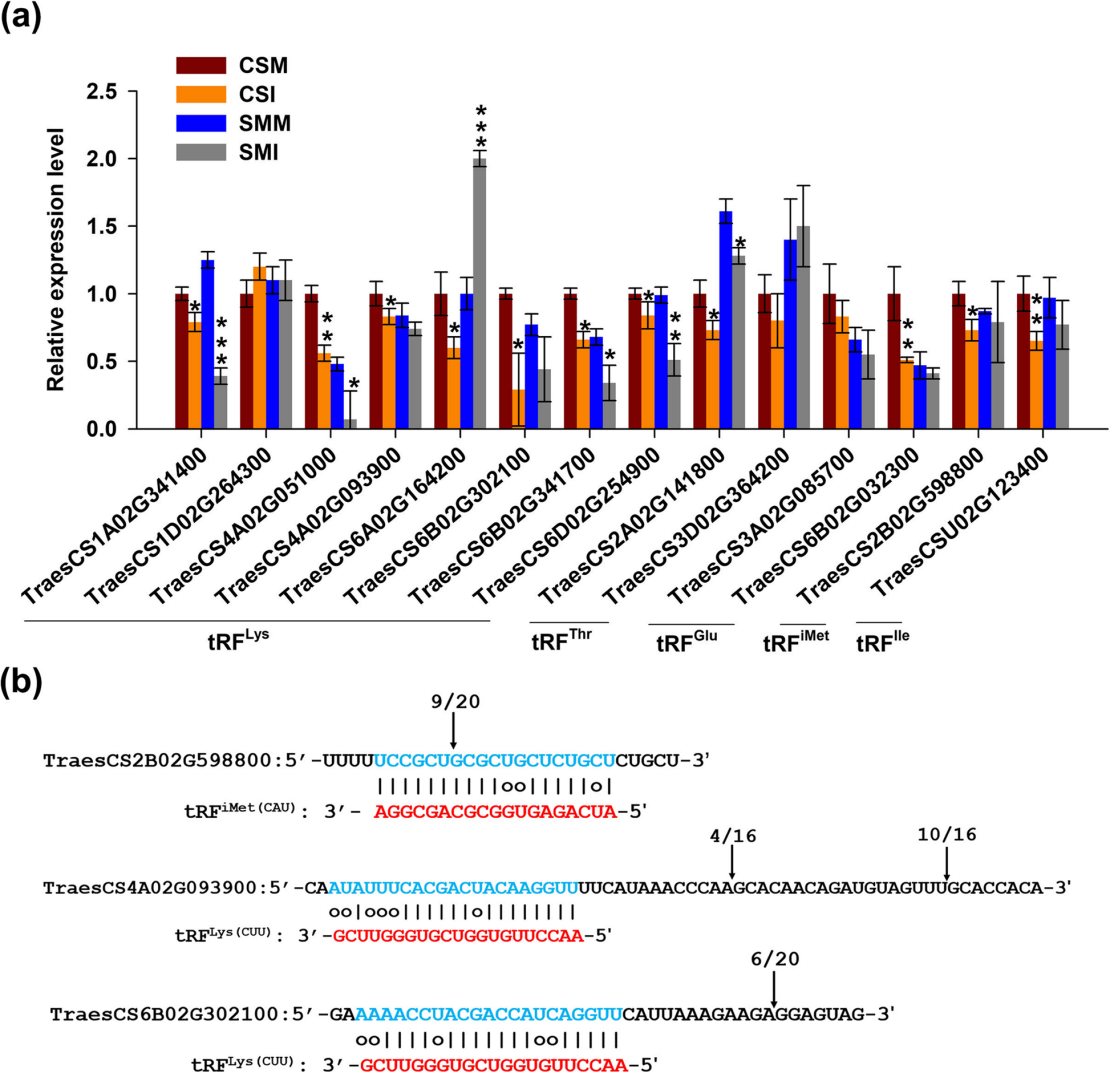
Experimental verification of predicted tRFs’ targets. (**a**) Relative expression levels of 12 target genes for the tRF^Lys^, tRF^Thr^, tRF^Glu^ and 2 hub target genes. Expressional values are shown as means ± standard errors (s.e.) over three replicates. The *TaActin* genes was used as a loading control. Student’s *t*-test was used to compare the difference between *F*. *graminearum* (CSI or SMI) and mock (CSM or SMM) inoculations of the same variety. ***, *P* < 0.001; **, *P* < 0.01; *, *P* < 0.0 5. (b) 5’ RACE validation of the target genes. Vertical arrows indicate the cleavage site and the frequency of clones

qRT-PCR was performed to validate the tRFs’ target genes predicted from RNA-sequencing data. Ten predicted targets of tRF^Lys(CUU)^ and tRF^Thr(CGU)^ were selected for qRT-PCR in the four experimental groups. The expressional patterns of the majority of the target genes were highly consistent with transcriptome data (Fig. 7a). To further determine whether the target genes were regulated by tRFs in *vivo*, 5’ RNA Ligase-Mediated Rapid Amplification of cDNA Ends (5’ RLM-RACE) was conducted to capture the degraded products of the target mRNAs, which validated one of the tRF^iMet^ targets and two of the tRF^Lys^ targets (Figure. 7b).

All of above results indicated that tRFs probably interfered with the normal cell metabolism through inhibiting their targets.

## Discussion

Transfer RNA (tRNA) was traditionally considered to be a hub adaptor that transfers amino acids and helps ribosomes to decode messenger RNA [57, 58]. Therefore, the damage of tRNA will be disastrous for a cell. Previous studies showed that tRNA can be cleaved by DICER1 in animal or RNase T2 in planta [26, 59–61]. The cleaved fragment can inhibit mRNA transcriptional level, resembling the miRNA-like mechanism [18]. According to our data in wheat, the total counts of tRFs were higher than those of miRNAs especially after being infected by *F*. *graminearum* (Fig. 1a). The majority of tRFs ranged from 18 to 21 nt in sizes, which were compatible with the lengths of miRNAs (Fig. S3). Therefore, tRFs are expected to play important roles in planta. The size distribution and the corresponding abundance also indicated that not every tRF plays function like miRNA, and the generation of tRFs was under control instead of random degradation (Fig. 1b) during *F. graminearum* infection process of wheat. Recent study showed that RNase T2 is involved in the response to pathogens [37], and an earlier study showed that RNase T2 was a major driver of tRFs biogenesis in plant [26]. Moreover, all of the *TaDCL1* family members were dramatically inhibited by *F. graminearum* infection (Fig. S6), indicating *TaDCL1* might not participate in the biosynthesis of tRF. The consistent expressional patterns between RNase T2s and tRFs suggests RNase T2 most likely mediated the biogenesis of tRFs when wheat is challenged by *F*. *graminearum* infection (Fig. S3, Fig. 4). According to the existing research, DON prevents the elongation of peptide chain on ribosome [4], which could be the main factor causing tRNA accumulation after *F. graminearum* infection. Due to the high resistance of SM to FHB, the number of *F. graminearum* infected spikelets in SM was much less than that of CS in the same time after inoculation, and DON content in SM was also much lower than that in CS, therefore, the abundance of tRF in SM was less than that in CS. In addition, the expression of RNase T2 was significantly higher in CS than that in SM (Fig. 4b). The positive correlation in expression abundance between RNase T2 and tRF suggested that the accumulation of tRFs was not only regulated by DON, but also was directly regulated by RNase T2.

Although a lot of tRFs induced by *F*. *graminearum* were wheat genotype-specific, especially in FHB-susceptible variety, the abundance of these specific tRFs were extremely low, most of which were less than 100 TPM (Table S3). Hence, the highly abundant and differentially expressed tRFs should be prioritized. But high abundance of tRFs doesn’t mean that they respond to *F*. *graminearum* infection, for instance, the most enriched tRF^Glu(CUC-1-1#3)^ did not have significant differences between *F*. *graminearum* and mock inoculations (Fig. 1d, Table S2, S4). Totally eight tRFs derived from RNA-Lys-CUU-12-1 (Table S4), and three of them, tRF^Lys(CUU-12-1 #1)^, tRF^Lys(CUU-12-1#2)^ and tRF^Lys(CUU-12-1#4)^, were significantly differentially expressed between *F. graminearum* and mock inoculations (Table 1). Although tRF^Lys(CUU-12-1#5)^ had the highest abundance, no significant differences were observed either between CS and SM or between *F. graminearum* and mock inoculations (Table S4). The mature sequences of the aforementioned four tRF^Lys^ were highly similar (Table S4.). Another interesting finding was that not every tRF identified here responded to *F*. *graminearum* infection, such as tRF^Asp^, tRF^Arg^, tRF^Gly^ and tRF^Ser^ (Table 1, Table S4).

The function of tRFs is one of the most important topics of interest. According to the published work, tRFs can bind to AGO protein to form RNA-induced silencing complex (RISC) to inhibit the targets by resembling miRNA function [62]. The majority of the predicted targets for induced tRFs were downregulated after *F*. *graminearum* invasion (Fig. 5a). These targets play pivotal roles in stress response, energy metabolism, cell component and protein digestion (Table 2, Fig. 5b-e). *TraesCS2A02G097900*, the target of tRF^Ala(UGC)^, was the key polymerase in DNA replication. *TraesCS2D02G337300* and *TraesCS2A02G325400* potentially targeted by tRF^iMet(CAU)^ regulate the protein biosynthesis as translation initiation factor 4B. tRNA-iMet-CAU transfers the initiation amino acid methionine, and it is the most important tRNA for protein biosynthesis. Here, we found the tRFs from tRNA-iMet-CAU might play a role in regulating initiation of translation based on the annotation of tRF^iMet^ targets, *TraesCS2D02G337300* and *TraesCS2A02G325400*, which encode translation initiation factor 4B. tRF^Lys(CUU)^ might inhibit the function of E3 ubiquitin-protein ligase by cleaving its target genes *TraesCS1A02G341400*, *TraesCS1D02G343700* and *TraesCS1B02G354100*, which play pivotal roles at the first step of protein degradation. ATP-binding cassette (ABC) transporter proteins are the important channel system for material communication in cytomembrane by carrying diverse substrates across cell membranes [63], which are the predicted targets of tRF^Cys(GCA)^ and tRF^His(AUG)^ based on our study.

Several genes were reported to be associated with disease resistance, such as *TraesCS7B02G416600*, *TraesCS2B02G045400*, *TraesCS6D02G041400* and *TraesCS7B02G437300*, which encodes disease-resistance protein RPM1 [64], RPP13-like protein [65], RPS2 [66], RPM13 [67], and they were the predicted targets of tRF^His(AUG)^, tRF^iMet(CAU)^, tRF^Lys(CUU)^, and tRF^Pro(CGG)^, respectively. Four hub targets of tRFs, *TraesCS2B02G598800*, *TraesCS6D02G254900*, *TraesCSU02G123400* and *TraesCS2A02G141800*, may play crucial roles in host resistance to pathogen attack (Fig. 6). *TraesCS2B02G598800* encodes polyadenylate-binding protein, which was associated with ribonucleic acid (RNA) stress granule (SG) pathways [68]. *TraesCS6D02G254900* encodes a histone-lysine N-methyltransferase SUVR5, which mediates H3K9me2 deposition and regulates gene expression in a DNA methylation-independent manner by regulating their chromatin and transcriptional state to rapidly adapt to environment or developmental cues [69]. *TraesCSU02G123400* encodes an adenylate kinase, which plays an important role in cellular energy homeostasis and in adenine nucleotide metabolism [70]. *TraesCS2A02G141800* plays a pivotal role at the level of protein transcription. In summary, these four hub genes regulate gene functions at DNA level (*TraesCS6D02G254900*), RNA level (*TraesCS2B02G598800*), protein level (TraesCS2A02G141800) and metabolic activity in cell (*TraesCSU02G123400*), and they were predicted to be targeted by corresponding tRFs when wheat was infected by *F*. *graminearum*.

The higher tRFs abundance in the susceptible variety CS may be related to its weak resistance to toxins, or the susceptible variety might induce *F. graminearum* to produce more DON toxin. The negative correlation of expression patterns between tRFs and disease resistance-related target genes indicates that these tRFs may contribute host susceptibility to *F*. *graminearum* by silencing their target genes. The resistance level of plant to the fungus is expected to be enhanced if the tRFs could be eliminated quickly or the targets could escape the suppression by tRFs-induced silencing.

## Conclusion

This is the first report of tRFs involved in wheat-*F*. *graminearum* interaction. One of the most important findings was that more tRFs were accumulated in FHB-susceptible variety CS than that in FHB-resistant variety SM. During infection of the wheat cells, *F*. *graminearum* secreted DON to attack wheat ribosome and inhibit the formation of peptide chain, consequently leading to accumulation of tRNA. RNase T2 was also induced by pathogen to degrade host tRNA to fragments of various sizes, and these tRFs could be assembled with AGO protein to form RNA-induced silencing complex (RISC) to inhibit the host gene expressions. Therefore, tRFs might negatively regulate wheat resistance to FHB by interfering with the normal cell metabolism, cell cycle, and some of the disease resistance genes at post transcriptional level. However, the relationships between tRFs and their targets need to be further deciphered.

## Methods

### Plant materials and *F. graminearum* inoculation experiment

Wheat varieties, Chinese Spring (CS)” and “Sumai3 (SM)”, were used in this study, which were kindly provided by Dr. Guihua Bai at Kansas State University, USA. CS is an FHB-susceptible variety [42, 43], and SM is a famous FHB-resistant variety which carries *Fhb1*, a major QTL conferring Type 2 resistance to FHB [44–46]. The seeds were surface sterilized by soaking in 70% ethanol for 10 min and then rinsed four times with sterile water. The sterilized seeds were sown in a mix of vermiculite and soil with a ratio of 1:3. The seedlings were grown in a condition-controlled phytotron about 3 weeks under 24 °C and 16 h of light / 8 h of dark cycle, and then were moved to a refrigerator at 4°C for vernalization for one month, and then the seedlings were moved back to the phytotron for growth.

Wheat spikes were inoculated with macroconidial spores of *F. graminearum* strain PH-1, which was donated by Dr. Bing Li at Zhengzhou University, China. Macroconidia were produced in mung bean broth following the protocol described by Bai et al .[47]. For each wheat variety, eight spikes were inoculated at early anthesis by injecting a 10 μL of the spore suspension (100 conidia μL^-1^) into the two bilateral florets of the fifth spikelet from the bottom of a spike. Mock inoculation was performed as a control, where a 10 μL of mung bean broth was used. The inoculated spikelets and their adjoined rachis were collected at 6 h, 12 h, 24 h, 2 day (d), 3 d, 4 d, 5 d, and 6 d post *F. graminearum* (CSI, SMI) and mock inoculations (CSM, SMM) respectively. Plants were grown in a condition-controlled phytotron under 28°C and 16 h of light / 8 h of dark cycle. Six independent biological replicates were conducted, with three biological replicates for sequencing and the remaining three for validation.

### RNA extraction

A mixed sample from the eight timepoints was prepared for RNA extraction. RNAiso plus reagent (TAKARA BIO INC, Shiga, Japan) was used for purification of the total RNA from the mixed samples according to the manual instructions. The tissue samples were ground in liquid nitrogen into powder and about 80 mg of the sample powder was then transfered into a 2 mL of tube with 1 mL of preheated RNAiso plus reagent and sufficient mixing using vortex oscillator. 200 μL of chloroform was added into the mixture. After being centrifuged at 12,000 × g for 10 min at 4 °C, 700 mL of the supernatant was transferred to a new 1.5 mL RNase free tube. The supernatant was mixed with an equal volume of isopropyl alcohol and placed at -20°C for 1 hour for precipitation. After that, the mixture was centrifuged at 12,000×g for 15 min at 4 °C and the supernatant was removed. After being washed with 1 mL of 75% ethanol, the RNA pellet was air-dried in the biosafety cabinet and was dissolved by 50 μL of DEPC-treated water. The quantification of the total RNA was assessed by NanoDrop 2000c spectrophotometer (Thermo Fisher Scientific, Lenexa, KS, USA), and RNA quality was checked using Agilent 2100 bioanalyzer (Thermo Fisher Scientific, MA, USA).

### Small RNA library construction

A small RNA library was prepared with 1 μg of the total RNA for each sample. The total RNA was purified by electrophoretic separation on a 15% urea denaturing polyacrylamide gel electrophoresis (PAGE) gel and the small RNA regions corresponding to 18-30 nt bands in the marker lane (14-30 ssRNA Ladder Marker, TAKARA) were excised and recovered. Then the small RNAs of 18-30 nt were ligated to adenylated 3’ adapters annealed to unique molecular identifiers (UMI), followed by the ligation of 5’ adapters. The adapter-ligated small RNAs were subsequently transcribed into cDNA by SuperScript II Reverse Transcriptase (Invitrogen, USA) and then several rounds of PCR amplification with PCR Primer Cocktail and PCR Mix were performed to enrich the cDNA fragments. The PCR products were separated by agarose gel electrophoresis with target fragments of 110-130 bp, and then purified by QIAquick Gel Extraction Kit (QIAGEN, Valencia, CA). The distribution of the fragment sizes in the library was checked using the Agilent 2100 bioanalyzer. The library was quantifed using real-time quantitative PCR (QPCR) (TaqMan Probe). The final ligated PCR products were sequenced using the BGISEQ-500 platform (BGI-Shenzhen, China).

### mRNA library construction

Oligo(dT)-attached magnetic beads were used to purify mRNA. The purified mRNA was fragmented into small pieces in a fragment buffer at appropriate temperature. Then first-strand cDNA was generated using random hexamer-primed reverse transcription, followed by a second-strand cDNA synthesis. Afterwards, A-Tailing Mix and RNA Index Adapters were added by incubating to end repair. The cDNA fragments obtained from previous step were amplified by PCR, and the products were purified by Ampure XP Beads, then were dissolved in EB solution. The product was validated on the Agilent Technologies 2100 bioanalyzer for quality control. The double stranded PCR products from the previous step were heated to be denatured and circularized by the splint oligo sequence to get the final library. The single strand circle DNA (ssCir DNA) was normalized as the final library. The final library was amplified with phi29 to make DNA nanoball (DNB), with more than 300 copies of one molecular, and the DNBs were loaded into a patterned nanoarray and single end 50 bases reads were generated on BGIseq500 platform (BGI-Shenzhen, China) [48].

### Data analysis

The raw sequencing data are called raw tags. The raw tags were processed following the next steps: (1) removing low quality tags; (2) removing tags with 5’ primer contaminants; (3) removing tags without 3’ primer; (4) removing tags without insertion; (5) removing tags with poly A. The processed reads longer than 18 nt were then mapped to the *F. graminearum* reference genome sequence (FungiDB) using Bowtie2 (v2.2.5) [49] with a parameter of “-v 0”. The reads unmapped to the *F. graminearum* sequences were subsequently mapped to the wheat reference genome sequence (EnsemblPlants 47 release) using Bowtie2 (v2.2.5) [49]. The reads perfectly mapped to the wheat genome sequence without any mismatches were further annotated and those perfectly matching transfer RNA (tRNA) sequences of *Triticum aestivum* (http://gtrnadb.ucsc.edu/GtRNAdb2/) were considered as wheat tRNA derived fragments (tRFs). For miRNA and sRNA, raw reads were filtered using the fastx_toolkit (http://hannonlab.cshl.edu/ fastx_toolkit/). The clean reads of 18-29 nt in length were mapped to a structural RNA (ribosomal RNAs, transfer RNAs, small nucleolar RNAs and small nuclear RNAs) database (http://rfam.xfam.org/). Then the unmapped reads were aligned to the *Triticum aestivum* genome (EnsemblPlants 47 release, https://plants.ensembl.org/Triticum_aestivum) using Bowtie. Relative abundance of unique wheat tRFs in each library was normalized to transcript per million reads (TPM).

For mRNA, the sequencing data was filtered with SOAPnuke (v1.5.2) [50] by (1) removing those reads containing sequencing adapter; (2) removing those reads with low-quality base ratio (base quality less than or equal to 5) more than 20%; (3) removing those reads with unknown base ('N' base) ratio more than 5%, afterwards clean reads were obtained and stored in FASTQ format. The clean reads were mapped to the *Triticum aestivum* reference genome (EnsemblPlants 47 release, https://plants.ensembl.org/Triticum_aestivum) using HISAT2 (v2.0.4) [51]. Bowtie2 (v2.2.5) was applied to align the clean reads to the reference gene, then the expression level of a gene was calculated by RSEM (v1.2.12) [52]. Differential expression analysis was performed using the DESeq2 (v1.4.5) [53] with Q value ≤ 0.05. GO (http://www.geneontology.org/) and KEGG (https://www.kegg.jp/) enrichment analysis of annotated different expressed gene were performed by Phyper (https://en.wikipedia.org/wiki/Hypergeometric_distribution) based on Hypergeometric test. The significant levels of terms and pathways were corrected by Q value with a rigorous threshold (Q value ≤ 0.05) by Bonferroni [54].

### Prediction of target genes

To identify the targets for the tRFs, psRNAtarget (http://plantgrn.noble.org/psRNA Target/), a plant miRNA target finder software web tool, was used. The parameters were set to *Triticum aestivum* reference genome to BLAST the target sites against the tRF sequences. To annotate the target genes for tRFs which were significantly differentially expressed, Blast to Gene Ontology (Blast2GO) (http://www.blast2go.com/b2glaunch tool) was used against the Nr database of National Center for Biotechnology Information (NCBI) (ftp://ftp.ncbi.nih.gov/blast/db/ ).

### qRT-PCR validation of tRFs

Stem-loop quantitative reverse transcription PCR (stem-loop qRT-PCR) [55] was performed to evaluate relative abundance of wheat tRFs. The specificity of stem loop RT-PCR for individual tRFs was confirmed by sequencing of the amplified fragments. *TaU6* gene was used as an internal reference to quantify the relative abundance of tRFs determined by stem-loop qRT-PCR from three technological replicates.

### 5’ RLM-RACE

A mixed sample of CSI was prepared for RACE. Total RNA was extracted and purified using the method described above. A SMARTer RACE 5’/3’ Kit (Takara Bio Inc., Kusatsu, Japan) was used to generate RACE products following the manufacturer’s protocol. The PCR products were cloned into the pEASY-Blunt3 vector (Transgen biotech, Beijing, China) and sequenced in GENEWIZ company (Suzhou, China). Gene-specific primers was listed in Table S7.

### tRNA structure draw

RNAfoldWebServer (http://rna.tbi.univie.ac.at//cgi-bin/RNAWebSuite/RNAfold. cgi) was used to draw the tRNA primary structures of tRFs.

### Statistical analysis

The charts in this study were drawn using GraphPad Prism5 and SigmaPlot 10.0 (Systat Software). SPSS19 was used for data analysis. One-way ANOVA and student’s *t* test were performed to generate *p* values. Heatmap, volcano plot and venn chart were produced by TBtools software [56]. STRING (version 11) was used to analyze the interaction between the target genes for tRFs in wheat.

## Supplementary Information

**Additional file 1: Figure S1. **FHB disease symptoms of Chinese spring (CS) and Sumai 3 (SM). (a) Photos were taken at 10 d after inoculation with *F. graminearum*. The arrow in yellow indicates the spikelet inoculated with *F. graminearum.* CSM, Chinese Spring (CS) with mock inoculation; CSI, CS with *F*. *graminearum* inoculation; SMM, Sumai3 (SM) with mock inoculation; SMI, SM with *F*. *graminearum* inoculation. Bar=2cm. (b) Number of diseased spikelets scored at 10 d after inoculation. Student’s *t*-test was used to compare the difference between CSI and SMI.

**Additional file 2: Figure S2.** Overview of tRFs and tRNA matching diagrams.

**Additional file 3: Figure S3. **Abundance and size of the wheat tRFs under mock and *F*. *graminearum* inoculations. The x-axis indicates the size of the major wheat tRFs ranging from 18 to 28 nt and the y-axis indicates the means of normalized tag counts of different sizes of the tRFs from small RNA-Seq.

**Additional file 4: Figure S4. **The expressional patterns of tRF^Glu^, tRF^Lys^ and tRF^Thr^ at different time points. (a), (b) and (c) show the expressional levels of corresponding tRF in time course by stem-loop-qRT-PCR. The x-axis represents the sample collecting time after inoculation with *F. graminearum*; TPI, time post inoculation. The y-axis represents the expressional level of the tRF at each time point. Expressional values are calculated in 2^-ΔΔCt^. The snRNA gene *TaU6* was used as a loading control. Similar results were obtained from three biological repeats.

**Additional file 5: Figure S5. **The expression profiles of *TaRNase T2* family members by RNA-Seq. Expression levels are shown as means ± standard errors (s.e.) over three biological replicates. Student’s *t*-test was used for difference analysis, ***, *P* < 0.001; *, *P* < 0.05.

**Additional file 6: Figure S6.**The expression profiles of *TaAGO1* and *TaDCL* family members by RNA-seq. Expression levels are shown as means ± standard errors (s.e.) over three biological replicates. Student’s *t*-test was used for difference analysis, ***, *P* < 0.001.

**Additional file 7: Table S1.** Statistics of sequencing libraries

**Additional file 8: Table S2.** Summary of the identified tRFs

**Additional file 9: Table S3.** Lists of specific tRFs in different groups

**Additional file 10: Table S4.** List of highly expressed tRFs

**Additional file 11: Table S5.** Summary of fragments derived from tRNA

**Additional file 12: Table S6.** The list of tRFs’ targets in wheat

**Additional file 13: Table S7.** The primers used in this study

**Additional file 14.**

## Data Availability

The data that support the findings of this study are available from the corresponding author upon reasonable request. All sequences generated by sequencing in this study can be found in the NCBI Short Reads Archive (SRA) BioProject PRJNA683746 (https://www.ncbi.nlm.nih.gov/sra/?term=PRJNA683746).

## Abbreviations

FHB: Fusarium head blight
tRNAs: transfer RNAs
tRFs: tRNA-derived fragments
DCL1: Dicer-like protein 1
miRNA: microRNA
RISC: RNA-induced silencing complex
CS: Chinese Spring
SM: Sumai3
DON: deoxynivalenol
QTL: quantitative trait loci
CSM: Chinese Spring inoculated with mock
CSI: Chinese Spring inoculated with *F. graminearum*
SMM: Sumai3 inoculated with mock
SMI: Sumai3 inoculated with *F. graminearum*
TPM: transcripts per million reads
DPI: days post inoculation
RNS: S-like RNases
