# Supplementary figures and images for: tRNA-derived fragments from wheat are potentially involved in susceptibility to Fusarium head blight
Source: BMC Plant Biol. 2022 Jan 3;22:3. doi: 10.1186/s12870-021-03393-9 (PMC8722339; doi:10.1186/s12870-021-03393-9)

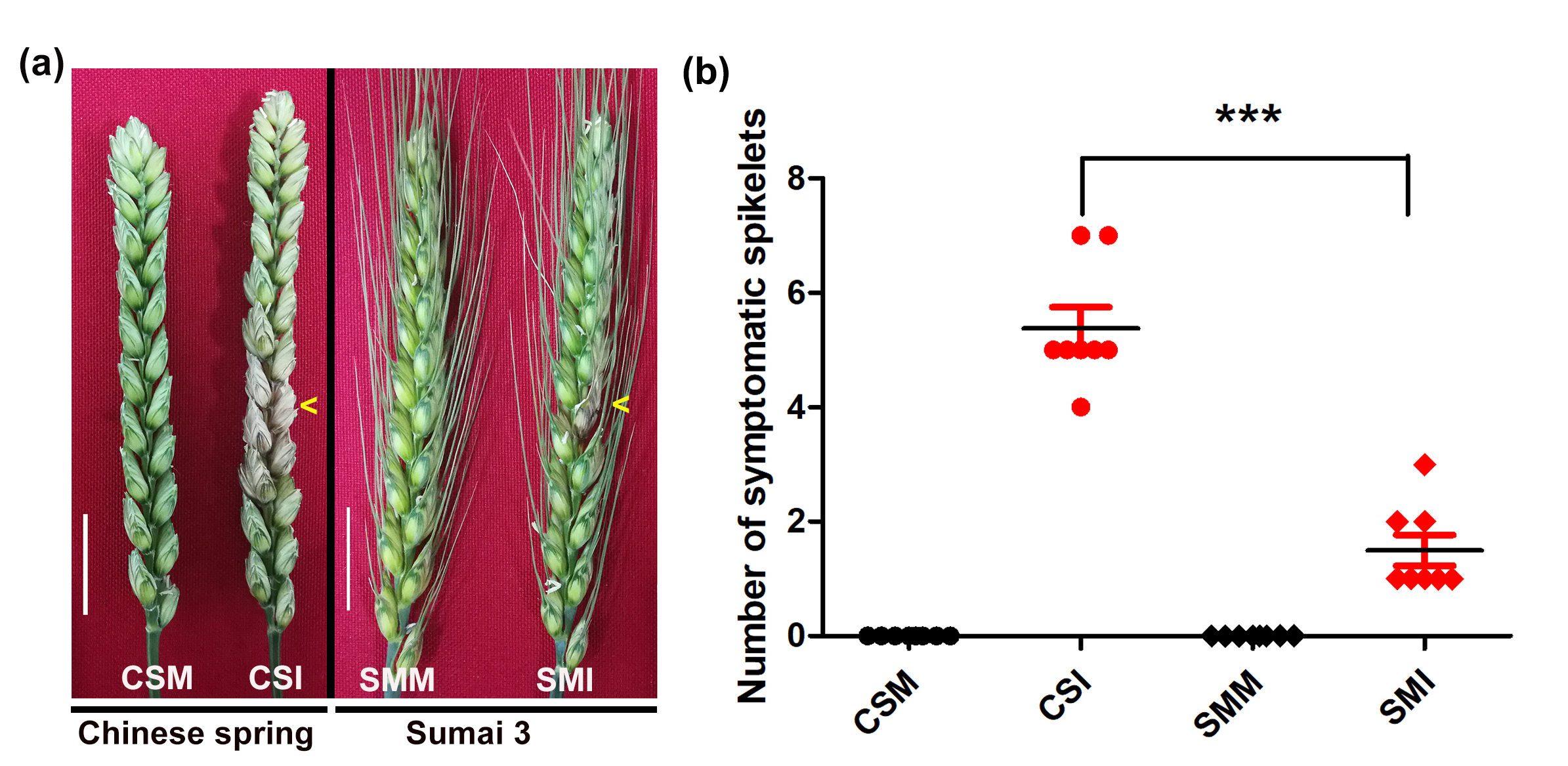

Supplement: Supplementary file 1 — Additional file 1: Figure S1. FHB disease symptoms of Chinese spring (CS) and Sumai 3 (SM). (a) Photos were taken at 10 d after inoculation with F. graminearum. The arrow in yellow indicates the spikelet inoculated with F. graminearum. CSM, Chinese Spring (CS) with mock inoculation; CSI, CS with F. graminearum inoculation; SMM, Sumai3 (SM) with mock inoculation; SMI, SM with F. graminearum inoculation. Bar=2cm. (b) Number of diseased spikelets scored at 10 d after inoculation. Student’s t-test was used to compare the difference between CSI and SMI. [file 12870_2021_3393_MOESM1_ESM.jpg]

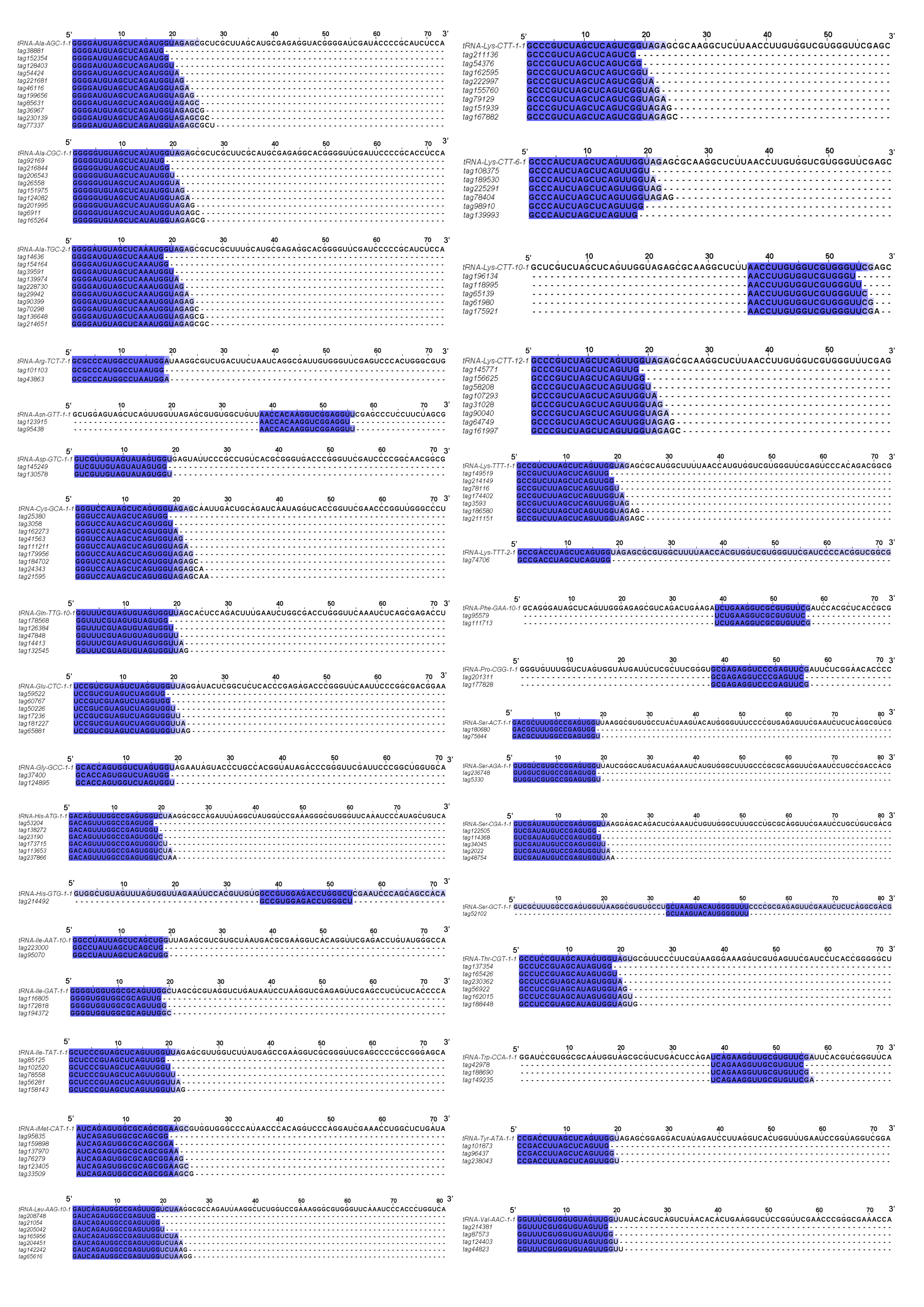

Supplement: Supplementary file 2 — Additional file 2: Figure S2. Overview of tRFs and tRNA matching diagrams. [file 12870_2021_3393_MOESM2_ESM.jpg]

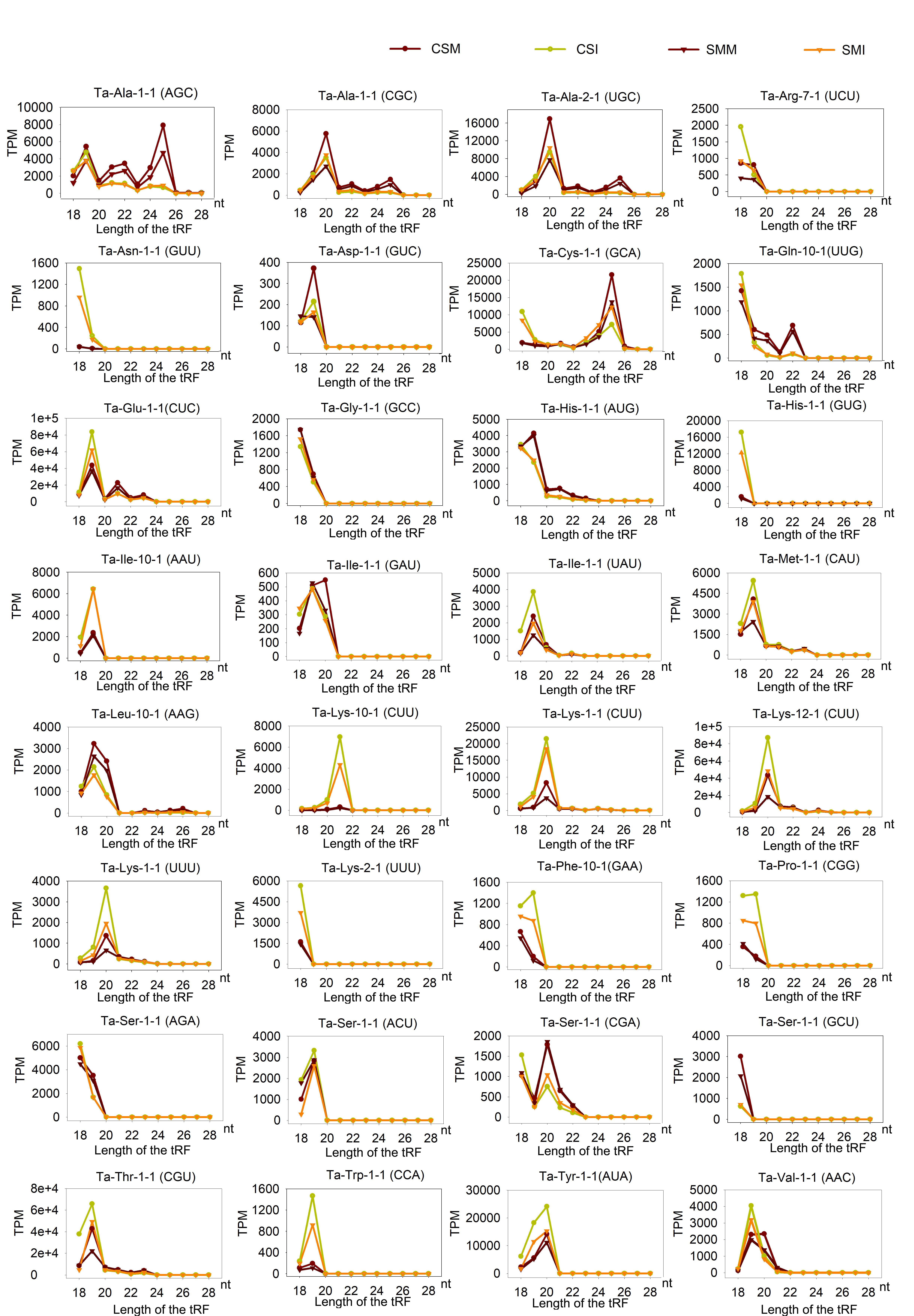

Supplement: Supplementary file 3 — Additional file 3: Figure S3. Abundance and size of the wheat tRFs under mock and F. graminearum inoculations. The x-axis indicates the size of the major wheat tRFs ranging from 18 to 28 nt and the y-axis indicates the means of normalized tag counts of different sizes of the tRFs from small RNA-Seq. [file 12870_2021_3393_MOESM3_ESM.jpg]

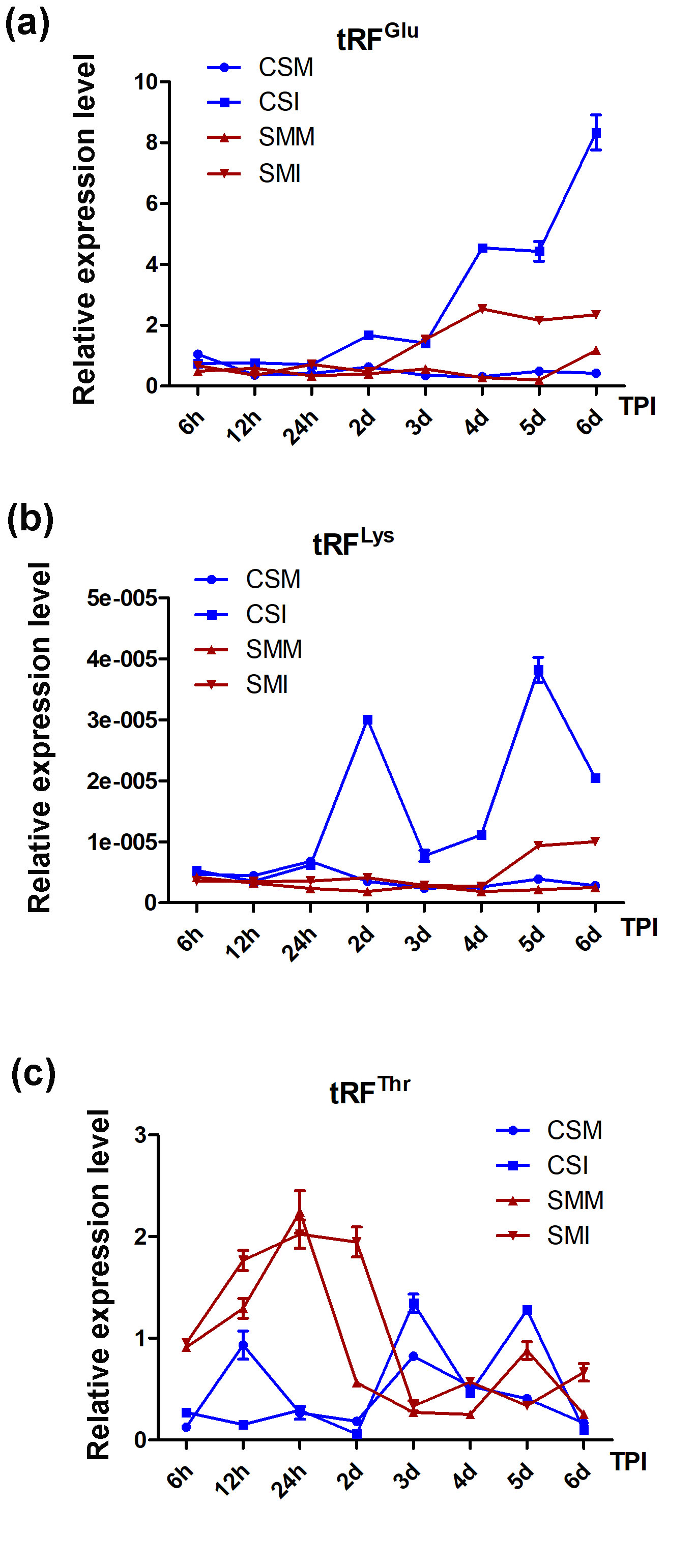

Supplement: Supplementary file 4 — Additional file 4: Figure S4. The expressional patterns of tRFGlu, tRFLys and tRFThr at different time points. (a), (b) and (c) show the expressional levels of corresponding tRF in time course by stem-loop-qRT-PCR. The x-axis represents the sample collecting time after inoculation with F. graminearum; TPI, time post inoculation. The y-axis represents the expressional level of the tRF at each time point. Expressional values are calculated in 2-ΔΔCt. The snRNA gene TaU6 was used as a loading control. Similar results were obtained from three biological repeats. [file 12870_2021_3393_MOESM4_ESM.jpg]

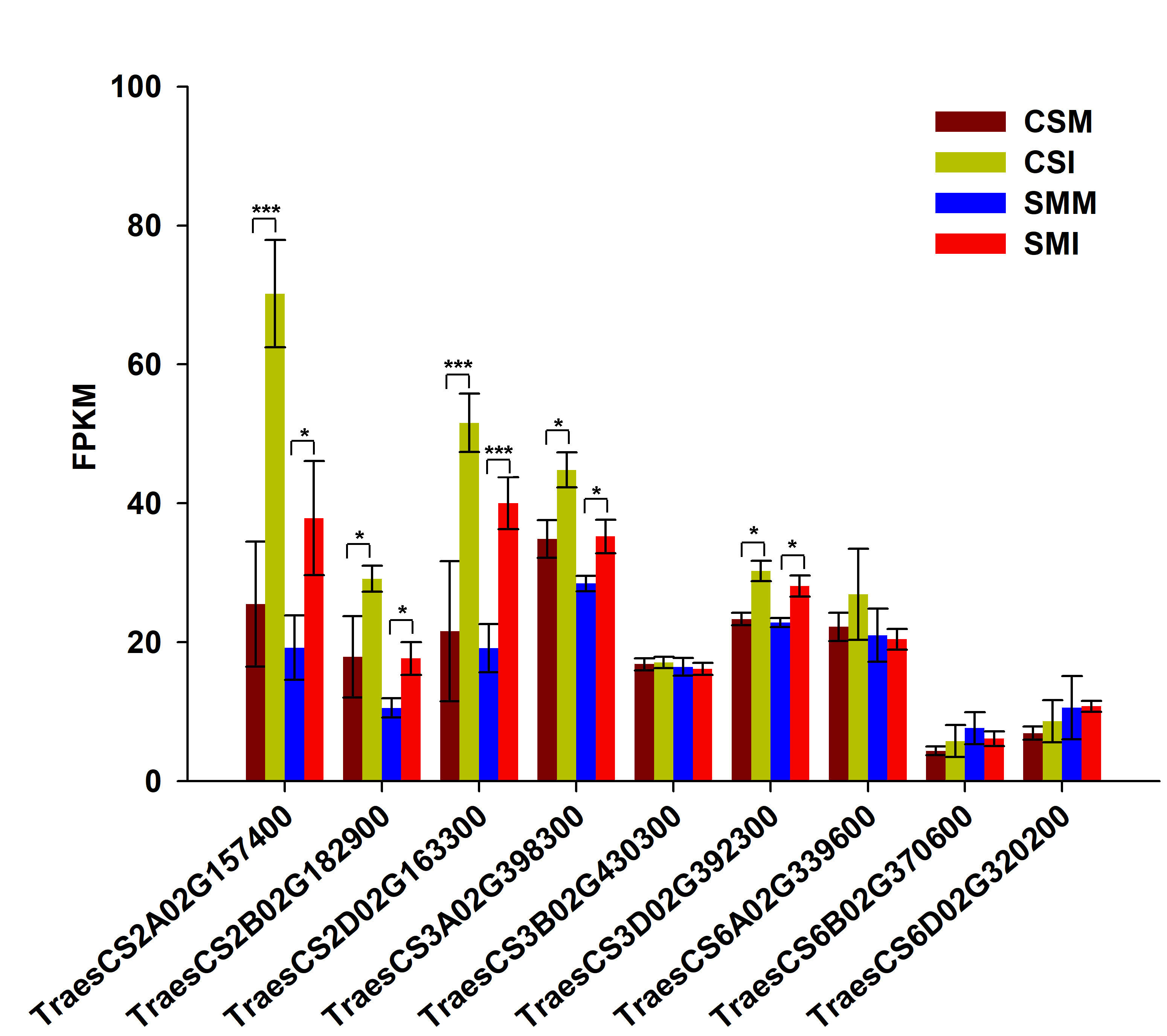

Supplement: Supplementary file 5 — Additional file 5: Figure S5. The expression profiles of TaRNase T2 family members by RNA-Seq. Expression levels are shown as means ± standard errors (s.e.) over three biological replicates. Student’s t-test was used for difference analysis, ***, P < 0.001; *, P < 0.05. [file 12870_2021_3393_MOESM5_ESM.jpg]

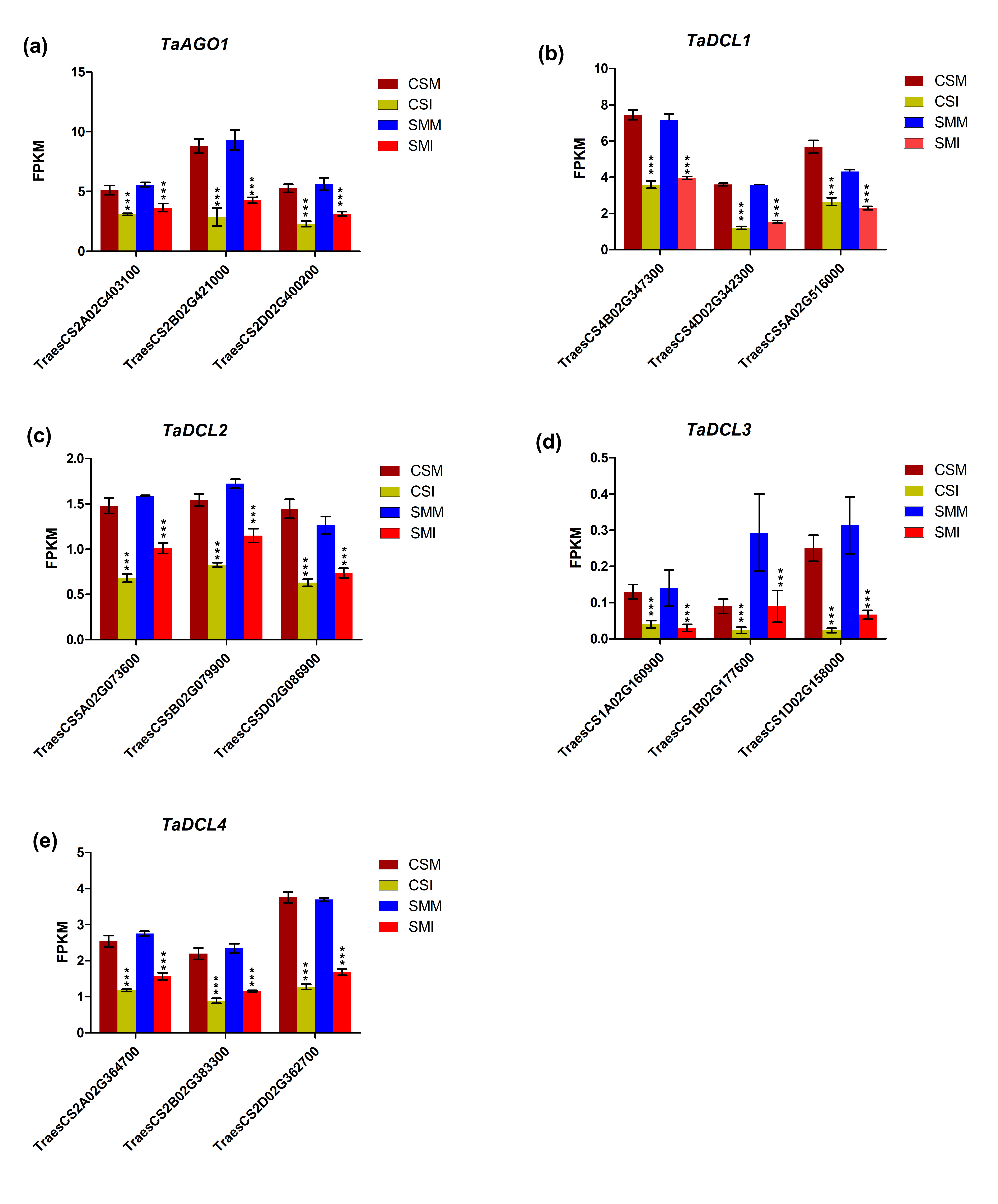

Supplement: Supplementary file 6 — Additional file 6: Figure S6.The expression profiles of TaAGO1 and TaDCL family members by RNA-seq. Expression levels are shown as means ± standard errors (s.e.) over three biological replicates. Student’s t-test was used for difference analysis, ***, P < 0.001. [file 12870_2021_3393_MOESM6_ESM.jpg]
